# Constrained proteome allocation affects coexistence in models of competitive microbial communities

**DOI:** 10.1038/s41396-020-00863-0

**Published:** 2021-01-11

**Authors:** Leonardo Pacciani-Mori, Samir Suweis, Amos Maritan, Andrea Giometto

**Affiliations:** 1 Dipartimento di Fisica e Astronomia “Galileo Galilei”, Università degli Studi di Padova, Via Francesco Marzolo 8, 35131 Padova, Italy; 2 Department of Physics, Harvard University, 17 Oxford St, Cambridge, MA 02138 USA; 3 School of Civil and Environmental Engineering, Cornell University, 220 Hollister Dr, Ithaca, NY 14853 USA

**Keywords:** Biodiversity, Microbial ecology, Microbial ecology, Bacterial physiology

## Abstract

Microbial communities are ubiquitous and play crucial roles in many natural processes. Despite their importance for the environment, industry and human health, there are still many aspects of microbial community dynamics that we do not understand quantitatively. Recent experiments have shown that the structure and composition of microbial communities are intertwined with the metabolism of the species that inhabit them, suggesting that properties at the intracellular level such as the allocation of cellular proteomic resources must be taken into account when describing microbial communities with a population dynamics approach. In this work, we reconsider one of the theoretical frameworks most commonly used to model population dynamics in competitive ecosystems, MacArthur’s consumer-resource model, in light of experimental evidence showing how proteome allocation affects microbial growth. This new framework allows us to describe community dynamics at an intermediate level of complexity between classical consumer-resource models and biochemical models of microbial metabolism, accounting for temporally-varying proteome allocation subject to constraints on growth and protein synthesis in the presence of multiple resources, while preserving analytical insight into the dynamics of the system. We first show with a simple experiment that proteome allocation needs to be accounted for to properly understand the dynamics of even the simplest microbial community, i.e. two bacterial strains competing for one common resource. Then, we study our consumer-proteome-resource model analytically and numerically to determine the conditions that allow multiple species to coexist in systems with arbitrary numbers of species and resources.

## Introduction

Microbes are among the most abundant life forms on Earth in terms of biomass [1]. They are found in almost every habitat of our planet, and continue to surprise us with their ability to survive in places that were thought to be inhospitable and barren. For example, microbial communities have been found in the deep terrestrial subsurface [2, 3], and it has been estimated that the first five kilometers beneath the Earth’s surface could be habitable for them [4]. Because of their ubiquity, microbial communities play fundamental roles in countless natural processes of vital importance, from the digestion and overall health of their host organism [5] to the regulation of bio-geochemical cycles [6, 7]. Despite their importance, however, we still know very little about the fundamental mechanisms that regulate microbial communities, partly because we are only able to grow in the lab a very small fraction of all the microbes found in nature [8], and partly because microbial communities are complex, non-linear systems [9] whose dynamics is difficult to predict. For these reasons, scientists from many disciplines have long been fascinated by the challenging theoretical questions posed by the study of microbial communities’ structure and dynamics, and serious efforts are being made to understand how competition [10–12] and metabolic interactions [13, 14] allow such systems to maintain the very high levels of biodiversity found in nature.

Recent experimental studies have shown that the structure and composition of microbial communities are tightly linked to the metabolism of the species that inhabit them [15, 16] (e.g., communities with different taxonomic compositions can nevertheless exhibit the same metabolic functional structure [17, 18]). We can therefore speculate that the ways with which microbes uptake and use different resources for growth and proliferation can affect the dynamics of an entire community. Resource uptake is constrained by the other functions that cells must perform to grow and proliferate, and the balance between such functions is governed by the allocation of the internal resources of the cell (e.g., the proteome, the set of proteins expressed by a cell) to different tasks. It is therefore important to understand how microbial community dynamics is influenced by the proteome allocation of its members, and new insights in this direction might help us make more powerful predictions of how microbial communities assemble and evolve [19, 20]. However, accounting for the dynamics of metabolism and gene expression of each species in a microbial community explicitly (e.g., via community flux balance analysis [21]) can be very challenging, and the large dimensionality of the mathematical models that attempt to do so poses limits to our understanding of the dynamics of microbial communities and of the fundamental properties that affect species coexistence.

Scott et al. [22] showed that, despite the complexity of bacterial metabolism, there are simple relationships that link the fraction of the proteome allocated for nutrient uptake and protein synthesis to the growth rate of bacteria grown in isolation, and that reducing these fractions by forcing cells to express a useless protein reduces their growth rate. Such relationships are very powerful because they describe how bacterial growth is influenced by proteome allocation and gene expression without requiring an explicit representation of the underlying molecular mechanisms. These relationships, which were also based on earlier observations by Schaechter et al. [23] on how the ribosomal component of the proteome of a microbial species scales with the growth rate, have recently been applied in many different contexts [24] and were instrumental in improving our knowledge of microbial metabolism, both experimentally [25] and computationally [26]. However, as the experiments by Scott et al. [22] were performed with single-species populations in exponential phase, it is still an open question if their approach can also be used to describe the population dynamics of different interacting microbial species competing for multiple resources.

In this work, we fill this gap by linking the results by Scott et al. [22] to one of the most widely adopted theoretical frameworks for modeling competitive ecosystems, MacArthur’s consumer-resource model [27–29], and use it to describe the dynamics of microbial species competing for one or more resources. MacArthur’s model describes how the population abundances of *N*_*S*_ species competing for a common pool of *N*_*R*_ resources change over time, and has been used in several recent studies [10–12, 30–32] to understand under which conditions multiple species can coexist while competing for few resources. These studies, however, did not account for the fact that proteome allocation constraints limit the rates at which microbes can uptake different resources, which, as shown here, affects the conditions that lead to the coexistence of multiple microbial species in competitive communities. We show that generalizing Scott et al.’s proteome-growth relationships and including them into a consumer-resource framework allows us to build a community dynamics model where all parameters can in principle be measured experimentally and have a precise biological interpretation. This “Consumer-Proteome-Resource” (CPR) model describes community dynamics at an intermediate level of complexity between classical consumer-resource models and biochemical models of microbial metabolism [21]. By adopting such an intermediate level of complexity and realism, we can take into account the dynamics of gene expression and microbial metabolism, while preserving analytical insights on the microbial community dynamics and identifying the key intracellular properties affecting species coexistence.

There have been attempts in the past at deriving models to describe the dynamics and/or structure of microbial communities by incorporating some insight into the metabolism of their species and the molecular aspects of their growth. One of the earliest and most notable efforts in this direction was performed by Droop [33], who developed a model that describes microalgal growth by taking into account intracellular quotas of the (single) supplied resource. In more recent times, the problem has been addressed by applying Flux Balance Analysis to genome-scale models in order to reveal how metabolic fluxes can influence community dynamics [34, 35]. This approach, however, leads to models that are extremely complicated and strongly dependent on the identity of the species in the community, since they require detailed knowledge of metabolic networks with hundreds of different reactions for every species, as well as the metabolic interactions among the members of the community. More recently, it has been shown that introducing some information on the metabolism of microbial species in models of community dynamics (without all the details that a Flux Balance Analysis model requires) can provide us with useful insights on the properties of the community [36, 37]. Our work sits conceptually in this latter context, but unlike what has already been done in this direction does not make assumptions on the metabolism of the species and relies on quantities (like the proteome fractions) that can be measured directly.

In the next section, we describe the CPR model for a general number of species/strains and resources. First, we review the proteome allocation framework of Scott et al. [22] and discuss how we generalize it to multiple resources. Second, we review the fundamental structure of consumer-resource models. Third, we construct our consumer-resource model which incorporates proteome allocation. Then, we consider the simplest implementation of an experimental microbial community, i.e. two *Escherichia coli* strains competing for glucose as the only carbon source, to illustrate that it is necessary to account for proteome allocation in consumer-resource models to describe the dynamics (and the conditions for coexistence) of even the simplest microbial community. The experiment described here constitutes a proof of the concept that one needs to account for proteome allocation dynamics when adopting consumer-resource theory to describe competitive microbial communities. Finally, we study (both analytically and numerically) the CPR model for communities composed of arbitrary numbers of species and resources to identify the conditions allowing the coexistence of multiple species in the community. A discussion section and some future perspectives conclude this work.

## Results

### Microbial proteome allocation

The phenomenological framework proposed by Scott et al. [22] prescribes that the proteome of a single microbial species growing on a single resource can be minimally divided into three sectors: one dedicated to nutrient uptake and metabolism (the “P-sector”), one dedicated to ribosomal proteins responsible for biomass production and growth (the “R-sector”), and a third one dedicated to housekeeping functions (the “Q-sector”), which was shown to be incompressible [22]. Naming *φ*^*P*^, *φ*^*R*^ and *φ*^*Q*^ the proteome fractions corresponding to these sectors, we must have *φ*^*P*^ + *φ*^*R*^ + *φ*^*Q*^ = 1 (since all proteome fractions must sum to one), and Scott et al. have shown that *φ*^*P*^ and *φ*^*R*^ are linear functions of the species’ growth rate *g*, i.e:

1a $$\varphi ^P = \frac{\rho }{{\bar \kappa ^n\left( c \right)}}g,$$

1b $$\varphi ^R = \frac{\rho }{{\kappa ^t}}g + \varphi ^0.$$

Here *ρ* is a conversion factor (equal to the ratio between the total mass of the ribosomal proteins and the total RNA mass of the cells) and $\bar \kappa ^n\left( c \right) = \kappa ^n \cdot r\left( c \right)$, where *r*(*c*) = *c*/(*K* + *c*) is the Monod function which encapsulates the dependence on the resource concentration *c*. Most of our results do not actually depend on the exact functional form of *r*(*c*), as long as *r*(*c*) is a monotonically increasing function that saturates for large values of *c* (see Materials and Methods). *K* is the half-saturation constant of the resource and *κ*^*n*^ is the “nutritional capacity” [22] of the (only) limiting resource. This parameter measures how much protein biomass is produced per unit ribosomal mass per unit time, and therefore depends on how much energy the resource contains and how efficiently the microbial species can metabolize it (see Supplementary Information and [22] for a molecular interpretation of *κ*^*n*^). The parameter *κ*^*t*^ is the “translational capacity” [22] of the microbial species, measuring how much protein biomass is produced per unit ribosomal mass per unit time; it is, therefore, a measure of how fast the microbial species expresses its genome to synthesize proteins. Finally, *φ*^0^ is the incompressible core of *φ*^*R*^, representing the fact that ribosomal proteins are present in the cells also when microbes are not growing. All these parameters involve the ribosomal mass of the microbial species because the measurements by Scott et al. [22] were done by assaying the RNA/protein ratio in exponentially growing *Escherichia coli*.

**Fig. 1:**
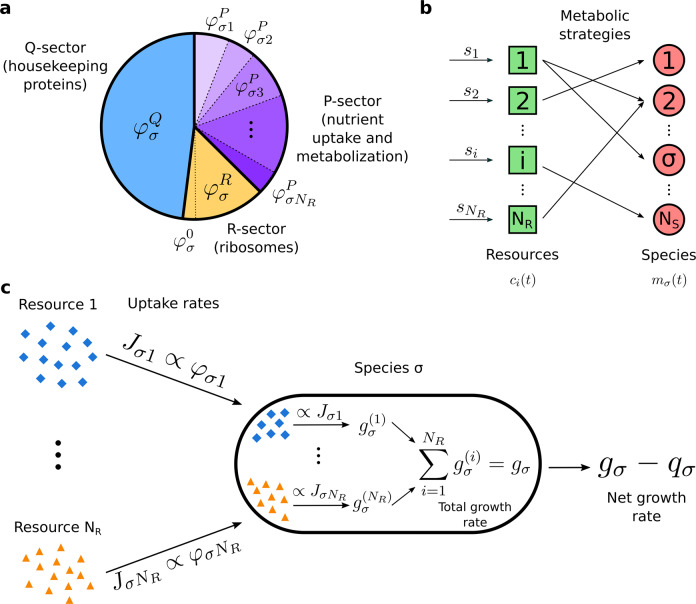
Assumptions of the CPR model. **a** Generalization of Scott et al.’s [22] proteome subdivision to the case of *N*_*R*_ resources: the proteomic sector allocated by species *σ* for nutrient uptake and metabolization is subdivided into smaller fractions $\varphi _{\sigma i} = \varphi _{\sigma i}^P$, each dedicated to a specific resource. **b** Schematic representation of a consumer-resource model with *N*_*R*_ resources and *N*_*S*_ species. In this framework, the concentrations *c*_*i*_ of the resources and the biomass densities *m*_*σ*_ of the species are described by systems of coupled differential equations. Resources are supplied with (constant) rates *s*_*i*_, and are uptaken by the species (arrows represent resource flows). The ways in which each species uptakes resources are encoded in the “metabolic strategies”. In our framework we are not considering the exchange of metabolic byproducts between species (i.e., cross-feeding). **c** Assumptions used to write the equations of the CPR model. Each species *σ* uptakes resource *i* with a rate *J*_*σi*_ proportional to the proteome fraction *φ*_*σi*_. Then, each resource contributes a growth term $g_\sigma ^{\left( i \right)}$ (proportional to the resource uptake rate) to the total growth rate. The net growth rate of species *σ* is the difference between the sum of these contributions and the maintenance cost *q*_*σ*_.

Scott et al.’s results apply to microbes growing on a single resource. We generalize their framework to a system with multiple species and resources as shown in Fig. 1a: indicating with $\varphi _{\sigma i}^P$ the proteome fraction allocated by species *σ* to the uptake and metabolization of resource *i*, the total proteome fraction allocated by species *σ* to nutrient uptake and metabolism is given by $\varphi _\sigma ^P = \mathop {\sum}\nolimits_{i = 1}^{N_R} {\varphi _{\sigma i}^P}$. To ensure that the sum of all the proteome fractions is equal to one we must have:

2 $$\varphi _\sigma ^Q + \varphi _\sigma ^R + \mathop {\sum}\limits_{i = 1}^{N_R} {\varphi _{\sigma i}^P} = 1.$$

This constraint represents the finiteness of a species’ proteome, i.e. the fact that each species in a community has a limited proteomic budget that can be spent for all the necessary biological functions: for example, if more proteins need to be produced for metabolizing complex substrates (i.e., if the nutrient fraction $\varphi _\sigma ^P$ increases), then a smaller part of the proteome will be available for biomass production (i.e., the ribosomal fraction $\varphi _\sigma ^R$ decreases). In order to achieve optimal growth, microbial species must balance this trade-off [22].

### Consumer-resource models

In Fig. 1b we show a schematic representation of the “classic” consumer-resource model. Within this framework, a community is a set of *N*_*S*_ species that can only uptake some (or all) of the *N*_*R*_ available resources. Species’ growth rates are determined by the types and the amount of resources they uptake, and are also regulated by a “maintenance cost”, representing the fact that species need to uptake a minimum amount of resources in order to survive. The resources, on the other hand, can be thought of as substrates that are supplied to the system with given (constant) rates *s*_*i*_, and they are uptaken by species in the community. Overall, the model describes explicitly the dynamics of both species and resources through equations with the following structure:

3a $$\dot m_\sigma = m_\sigma \left( {g_\sigma - q_\sigma } \right) \qquad \quad \sigma = 1, \ldots ,N_S,$$

3b $$\dot c_i = s_i - \mathop {\sum }\limits_{\sigma = 1}^{N_S} J_{\sigma i}m_\sigma \qquad \quad i = 1, \ldots ,N_R,$$

where *m*_*σ*_ is the biomass density of species *σ* and *g*_*σ*_ is its growth rate. The parameter *q*_*σ*_ is a maintenance cost, due to the fact that each species requires a minimum amount of energy per unit time to survive without growing. Finally, *c*_*i*_ is the density of resource *i*, *s*_*i*_ is the (constant) resource supply rate, and *J*_*σi*_ is the rate at which species *σ* uptakes resource *i* per unit biomass. The ways in which species uptake the available substrates are encoded in *J*_*σi*_ with parameters that in the literature are called “metabolic strategies” or “resource preferences”. In particular, consumer-resource models are generally setup so that *J*_*σi*_ ∝ *α*_*σi*_, with ${\vec{ \alpha}}_\sigma = \left( {\alpha _{\sigma 1}, \ldots ,\alpha _{\sigma N_R}} \right)$ the metabolic strategy (or resource preference) of species *σ*. Therefore, in the consumer-resource framework the interactions between species are indirect and mediated by the abundance of resources and the species’ resource preferences. Other types of direct inter-specific interactions (like cross-feeding through the exchange of metabolic byproducts), though undoubtedly important in natural microbial ecosystems, are not addressed in this work.

### The consumer-proteome-resource model

Here, we incorporate proteome allocation constraints into consumer-resource models and show that proteome fractions allocated to the uptake of different resources must vary with time as resource concentrations vary. Figure 1c depicts schematically the assumptions underlying the CPR model. Each species *σ* uptakes resource *i* with a rate *J*_*σi*_ that is proportional to the proteome fraction $\varphi _{\sigma i}^P$. Then, resource *i* accounts for a growth term $g_\sigma ^{\left( i \right)}$ proportionally to the uptake rate *J*_*σi*_. For our purposes, we assume that all resources in the system are substitutable, so that they can be used interchangeably and we can write the total growth rate *g*_*σ*_ of a given species as the sum of all the terms $g_\sigma ^{\left( i \right)}$. This assumption is consistent with previous works [38, 39] that considered the proteome allocation introduced by Scott et al. [22] in systems with two substitutable resources. Eventually, we obtain the following mathematical model (see Materials and Methods for the detailed derivation):

4a $$\dot m_\sigma = m_\sigma \left[ {\mathop {\sum}\limits_{i = 1}^{N_R} {\frac{{\kappa _i^n}}{{\rho _\sigma }}} r_i\left( {c_i} \right)\varphi _{\sigma i} - q_\sigma } \right],$$

4b $$\dot c_i = s_i - \xi _ir_i\left( {c_i} \right)\mathop {\sum}\limits_{\sigma = 1}^{N_S} {m_\sigma } \varphi _{\sigma i},$$

4c $$\mathop {\sum}\limits_{i = 1}^{N_R} {\varphi _{\sigma i}} \left[ {1 + \frac{{\kappa _i^n}}{{\kappa _\sigma ^t}}r_i\left( {c_i} \right)} \right] = {\Phi}_\sigma ,$$

where we have written $\varphi _{\sigma i} = \varphi _{\sigma i}^P$ for simplicity. The parameter *ξ*_*i*_ can be interpreted as the maximum catalytic rate of the enzyme used to metabolize resource *i*, and Φ_*σ*_ is the total proteome fraction allocated by species *σ* for metabolism and biomass synthesis, which is fixed as shown by Scott et al. [22]. These equations have the traditional structure of a consumer-resource model given by Eqs. (3a) and (3b), but with the added merit of describing population dynamics using parameters and variables that have a precise biological meaning at the intracellular scale of the system and that can in principle be measured experimentally [22]. For a species growing on a single resource, the parameters that are most easily measured experimentally are the per-biomass resource uptake rate *ξr*(*c*)*φ*_*σ*_ and the yield (expressed as biomass per grams of resource), which in our framework is given by *Y* = *κ*^*n*^/*ρ*ξ (see Supplementary Information).

Notice that the metabolic strategies in our framework correspond to the proteome fractions *φ*_*σi*_. If we interpreted the *φ*_*σi*_ as fixed parameters, the CPR model would be placed within the field of classic substitutable consumer-resource theory. However, we show below that  the proteome fractions *φ*_*σi*_ are actually dynamical variables that vary according to the concentration of resources, and thus the CPR model constitutes a generalization of classic consumer-resource theory with substitutable resources, based on experimental evidence of microbial proteome allocation and growth. In the CPR model the proteome fractions are subject to the constraint encoded by Eq. (4c), which derives from the proteome finiteness given by Eq. (2). The expression of this constraint is significantly different from other ones that have been studied in the consumer-resource framework [40]. Posfai et al. [31], for example, considered a classic consumer-resource model with fixed metabolic strategies, and a metabolic constraint that in our notation would read $\mathop {\sum}\nolimits_{i = 1}^{N_R} {\varphi _{\sigma i}} = \Phi$, where the sum does not depend on the resource concentrations through *r*_*i*_(*c*_*i*_), and it is assumed that Φ_*σ*_ = Φ for all *σ* (i.e., the value of Φ_*σ*_ is exactly the same for all species). Such a model, however, cannot reproduce the fact that microbial species vary their metabolic strategies with time according to the concentration of resources, and the constraint $\mathop {\sum}\nolimits_{i = 1}^{N_R} {\varphi _{\sigma i}} = \Phi$ does not account for the fact that, as a species invests more resources in nutrient uptake and metabolization (the *φ*_*σi*_) to achieve a higher growth rate, such an investment must be balanced by an increased investment in ribosomal proteins (the $\varphi _\sigma ^R$), both of which are constrained by the finiteness of the proteome.

The proteome finiteness constraint, as encoded by Eq. (4c), yields one important consequence that has important repercussions on the properties the CPR model. In particular, it implies that the proteome fractions *φ*_*σi*_ *cannot* be fixed parameters, but must change as the resources’ concentrations *c*_*i*_ change, and therefore they must be dynamical variables. This can be easily seen by considering a system with only one resource, for which Eq. (4c) reads

5 $$\varphi _\sigma \left[ {1 + \frac{{\kappa ^n}}{{\kappa _\sigma ^t}}r\left( c \right)} \right] = {{\Phi}}_\sigma ,$$

and thus the *φ*_*σ*_ *must* change as functions of the resource concentration:

6 $$\varphi _\sigma = \frac{{{{\Phi }}_\sigma }}{{1 + \frac{{\kappa ^n}}{{\kappa _\sigma ^t}}r\left( c \right)}}.$$

In particular, *φ*_*σ*_ must *decrease* as the resource concentration *c increases* (recall that *r*(*c*) is a monotonically increasing function). This occurs because if, for example, the available resource becomes scarce, cells will need to produce more catabolic proteins to meet their energy requirements. In the presence of multiple resources, the proteome finiteness constraint of Eq. (4c) implies that if the concentration of one resource *c*_*j*_ decreases, then either *φ*_*σj*_ or some of the *φ*_*σi*_ with *i* ≠ *j must* increase to satisfy the constraint, since Φ_*σ*_ is constant. Thus, it is *necessary* to introduce some form of dynamics on the proteome fractions that each species allocates for nutrient uptake and metabolization. This observation should not come as a surprise, given that microbes are known to adapt their proteome allocation and metabolic strategies according to which resources are available. Our approach is to require that all *φ*_*σi*_ evolve dynamically with a characteristic timescale to maximize the instantaneous growth rate of species *σ* in an adaptive process, while ensuring that the proteome finiteness constraint is satisfied at all times. The model equations and the mathematical details are discussed in the Materials and Methods.

### Experimental example of the influence of proteome allocation on population dynamics

**Fig. 2:**
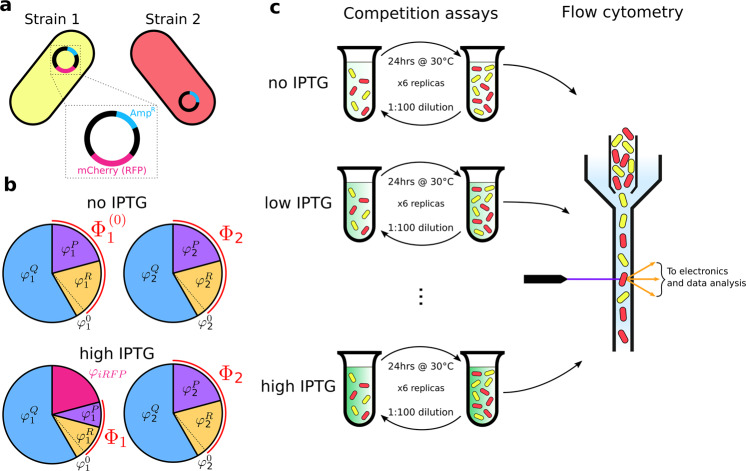
Schematic representation of the experiment. **a** Two E. coli strains were used: strain 1 constitutively expresses a yellow fluorescent protein (mVenus) and carries a plasmid with the ampicillin resistance cassette (cyan Amp^R^ in the plasmid magnification) and a red fluorescent protein (RFP), mCherry (magenta), under the control of the trc promoter, an hybrid of the trp and lac promoters. Strain 2 constitutively expresses a red fluorescent protein (mKate2Hyb) and carries a plasmid with the ampicillin resistance cassette. **b** Proteome allocation of the two strains at different concentration of IPTG in the medium. When strain 1 grows in the presence of IPTG, a fraction *φ*_*iRFP*_ of the strain’s proteome is allocated for the expression of the RFP mCherry, thus reducing the fraction Φ_1_ allocated for metabolism and growth. The proteome allocation of strain 2, instead, is not affected by the presence of IPTG. **c** The two strains were co-cultured in minimal medium at different IPTG concentrations, they were diluted daily into fresh medium and their relative abundance was measured at every transfer via flow cytometry.

Traditional consumer-resource models do not account explicitly for proteome allocation to different tasks and assume that metabolic strategies are fixed with time. Here, we show experimentally that it is necessary to take into account proteome allocation within consumer-resource models to reproduce the dynamics of even the simplest competitive community, i.e. two species competing for one common resource. We competed experimentally two strains of *E. coli* grown in a liquid minimal medium with glucose as the sole carbon source, transferring a fraction of the community to fresh medium daily and measuring the relative abundance of the two strains at each transfer (see Materials and Methods). The two strains had the same genetic background and expressed constitutively from their genome two different fluorescent proteins, which allowed us to measure their relative abundance via flow cytometry. We introduced in strain *σ* = 1 a plasmid containing a Red Fluorescent Protein (RFP) whose expression could be controlled by adding to the medium Isopropyl *β*-D-1-thiogalactopyranoside (IPTG, a molecular mimic of allolactose that cannot be metabolized by *E. coli*). Thus, by varying the concentration of IPTG in the medium we could vary the proteome allocation of strain 1 by forcing it to produce a useless protein. We performed competition experiments at different concentrations of IPTG, measured the fluorescent protein production rates at these concentrations, and computed the selective advantage of strain 1 over strain 2, a measure for the difference in reproductive fitness between the two strains defined as:

7 $$S: = \frac{d}{{dt}}{\mathrm{ln}}\frac{f}{{1 - f}},$$

where *f* is the relative abundance (or frequency) of strain 1, i.e. *f* = *m*_1_/(*m*_1_ + *m*_2_). The experiment is sketched in Fig. 2.

**Fig. 3:**
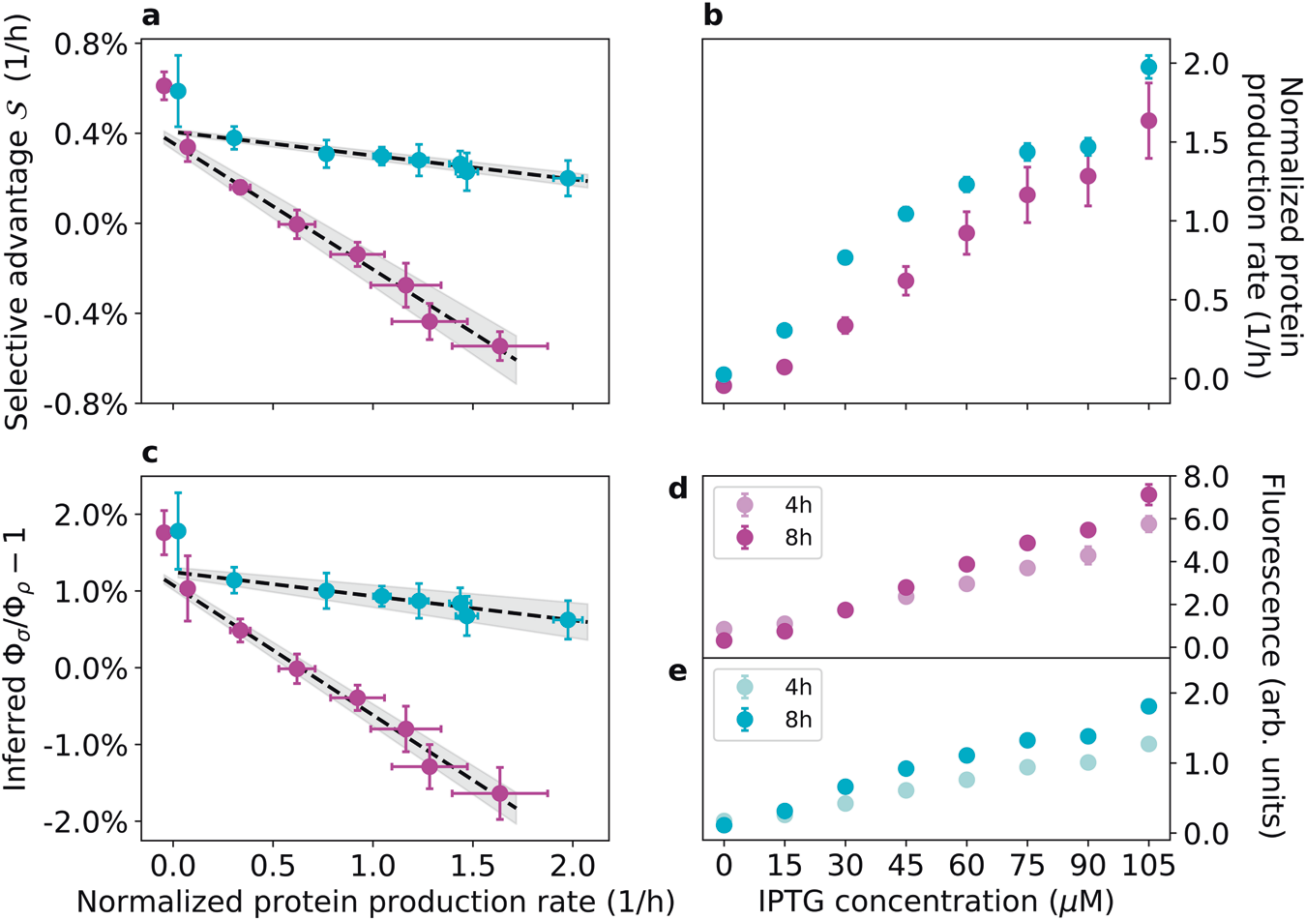
Experimental results. Magenta points represent data from experiments with strains 1 and 2. Cyan points represent data from experiments with strains 3 and 4, where strain 3 expresses constitutively mKate2Hyb and the IPTG-inducible Venus yellow fluorescent protein (YFP) and strain 4 expresses mVenus constitutively (see Materials and Methods and Fig. S.1). Error bars represent two standard deviations. Note that the normalized protein production rates are not directly comparable across magenta and cyan points (see Materials and Methods). **a** The experimental selection coefficient *S* (*y* axis) decreases linearly with the normalized production rate of the inducible protein, measured as the temporal variation of the mean cell fluorescence signal at different concentrations of the inducer IPTG, accounting for dilution of such protein via cell division (see Materials and Methods). The gray band represents the 68% confidence interval of the linear fit. The time series of ln[*f*/(1 − *f*)] for the two experiments are reported in Figs. S.19 and S.20. **b** Induced protein production rates as functions of IPTG concentration (see Materials and Methods). **c** Inferred values of the ratios Φ_1_/Φ_2_ and Φ_3_/Φ_4_ (minus one) as functions of the induced protein production rate (normalized). Also shown are the linear fits of the data with their 68% confidence interval. **d** Mean (induced) red fluorescence of strain 1 at 4 h and 8 h after inoculation in the conditions used for our experiment (see Materials and Methods). **e** Mean (induced) yellow fluorescence of strain 3 at 4 h and 8 h after inoculation in the conditions used for our experiment (see Materials and Methods).

Figure 3a (magenta data points) shows that the selective advantage *S* decreased linearly with the production rate of the IPTG-inducible RFP of strain 1 over a broad range or RFP production rates (the mean cell’s fluorescence measured after 8 h at 105 μM IPTG is 22 times higher than at 0 μM IPTG, Fig. 3d), which are proportional to *φ*_*iRFP*_. In the absence of IPTG and at low concentrations of it, strain 1 outcompeted strain 2 (*S* > 0). At an IPTG concentration of ~30 μM, the two strains coexisted by maintaining a stable relative fraction for the duration of the experiment. At IPTG concentrations larger than 30 μM, strain 1 was outcompeted by strain 2 (i.e., *S* < 0). This experiment illustrates that, in the presence of the same concentration of a single resource, manipulating the proteome allocation of one of the two strains results in different outcomes for their competition dynamics. Consumer-resource theory, which neglects proteome allocation dynamics, would not be able to predict competition dynamics in these settings.

Figure 3 also shows the results of a second experiment performed with two different strains (cyan data points). These strains had different fluorescent protein combinations with respect to strains 1 and 2 (see Materials and Methods and Fig. S.1): strain 3 expressed constitutively a red fluorescent protein (mKate2Hyb) and carried a plasmid with an IPTG-inducible yellow fluorescent protein (Venus YFP), while strain 4 expressed constitutively the yellow fluorescent protein mVenus (see Materials and Methods). Also in these independent sets of experiments, the selective advantage decreased linearly as the protein production rate was increased over a broad range (the mean cell’s fluorescence measured after 8 h at 105 μM IPTG was 16 times higher than at 0 μM IPTG, Fig. 3e). In this case, strain 3 always outcompeted strain 4, even at high concentrations of IPTG. This may be explained by the fact that the two proteins expressed by strains 1 and 3 have a different fitness cost (see Supplementary Information for more details).

It is natural to ask whether the CPR model can reproduce the results of our experiment. Applying the CPR framework to such a simple community, using assumptions consistent with our experimental settings (e.g., the fact that the strains are grown in medium-rich conditions, and that they share the same genetic background), leads to the prediction that the selective advantage *S* of strain 1 over strain 2 is given by (see Materials and Methods):

8 $$S = \frac{d}{{dt}}{\mathrm{ln}}\frac{f}{{1 - f}} \propto {\Phi}_1 - {\Phi}_2.$$

The same result could be obtained by assuming that the findings of Scott et al. [22] on how the exponential growth rate in isolation depends on proteome allocation can be applied to our experiment, in which cells were grown in co-culture dilution experiments and were not always in exponential phase. According to Eq. (8), the ratio between the relative abundances of the two strains decreases or grows exponentially with time, depending on the sign of Φ_1_ − Φ_2_, which then sets the outcome of competition: for example, if Φ_2_ > Φ_1_ (i.e., strain 2 allocates a larger fraction of its proteome to metabolism and biomass production than strain 1) then *S* < 0 and strain 2 outcompetes strain 1. Coexistence between the two strains is possible uniquely when Φ_1_ = Φ_2_ and thus *S* = 0. The system, therefore, exhibits two regimes where only one of the two strains survives (competitive exclusion), separated by the coexistence point Φ_1_ = Φ_2_. Equation (8) thus connects a well known concept of population genetics, the selective advantage in exponentially growing populations, with the differential proteome allocation Φ_1_ − Φ_2_ between microbial strains.

In our experiment, we forced strain 1 to produce a useless RFP at different rates depending on the IPTG concentration. Indicating with *φ*_*iRFP*_ the fraction of proteome allocated by strain 1 to the synthesis of the IPTG-inducible RFP (proportional to the fluorescent protein production rate), the proteome fraction allocated for nutrient uptake and growth is given by ${\Phi}_1 = {\Phi}_1^{\left( 0 \right)} - \varphi _{iRFP}$ (with ${\Phi}_1 = {\Phi}_1^{\left( 0 \right)}$ in the absence of IPTG). Thus, the selective advantage *S* is predicted to decay linearly with *φ*_*iRFP*_ as *S* = *α* − *β* · *φ*_*iRFP*_ with *α* and *β* positive constants (see the Materials and Methods section for all details and the explicit expression of *S* in this case). This prediction is thus consistent with the experimental observation of a linear decrease of *S* with the fluorescent protein production rate.

### Coexistence of multiple species in the consumer-proteome-resource model

We now analyze the CPR model in the general case of multiple species and multiple resources both analytically and numerically, to provide some insights into the conditions required for the coexistence of all species in the community. Specifically, we look for stationary solutions where *all* species have non-null biomass densities. Doing so yields two necessary conditions for the coexistence of all species (see the Materials and Methods for all detailed expressions and computations). The first condition, which holds when there are more species than resources in the system (*N*_*S*_ > *N*_*R*_), is that the maintenance cost *q*_*σ*_ of species *σ* must be proportional to the total proteome fraction allocated for metabolism and growth, i.e. *q*_*σ*_ ∝ Φ_*σ*_, with a species-dependent proportionality constant. This requirement is biologically reasonable, since allocating a larger fraction of the proteome to such functions requires additional energy to synthesize the necessary proteins. The condition is also required for all species to coexist if there are fewer species than resources (*N*_*S*_ ≤ *N*_*R*_) and all proteome fractions at stationarity $\varphi _{\sigma i}^ \ast$ are larger than zero. If, instead, there are fewer species than resources (*N*_*S*_ ≤ *N*_*R*_) and some proteome fractions at stationarity are equal to zero, it is possible to find particular solutions for which all species coexist, without requiring *q*_*σ*_ ∝ Φ_*σ*_. This happens, for example, when *N*_*S*_ ≤ *N*_*R*_ and the vectors ${\vec{\varphi}}_\sigma ^ \ast = \left( {\varphi _{\sigma 1}^ \ast , \ldots ,\varphi _{\sigma N_R}^ \ast } \right)$ are non-overlapping (i.e., ${\vec{\varphi}}_\sigma ^ \ast \cdot {\vec{\varphi}}_\rho ^ \ast = 0$ for *σ* ≠ *ρ*), which means that each species uses resources that are not used by other species. Further details can be found in the Materials and Methods.

**Fig. 4:**
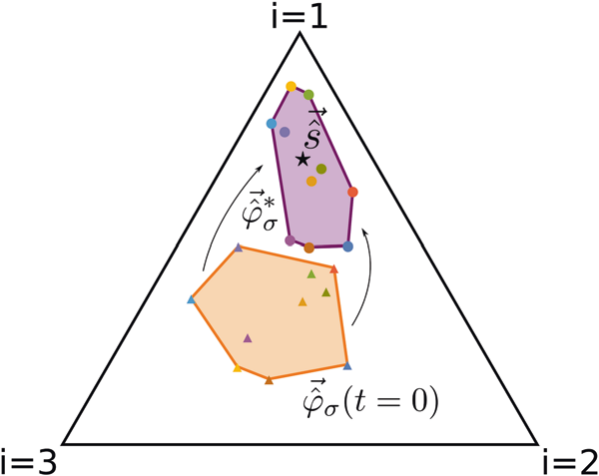
Graphical representation of the second condition necessary for coexistence. Here we consider a system with *N*_*S*_ = 10 species and *N*_*R*_ = 3 resources (for ease of representation). In this case, the system can be represented on a bi-dimensional simplex (i.e., a triangle) where each vertex corresponds to one of the available resources. On this simplex, we can draw the rescaled nutrient supply rate vector $\vec {\hat s}$ (black star) and the rescaled initial proteome fractions $\vec {\hat \varphi } _\sigma \left( {t = 0} \right)$ allocated by the species to the uptake and metabolism of the resources (colored triangles); their convex hull is drawn in orange. We have also drawn the stationary values $\vec {\hat \varphi } _\sigma ^ \ast$ of the proteome fractions (colored circles), and their convex hull is drawn in purple. In this representation, if $\vec {\hat s}$ lies on one on the sides of the simplex, it means that only two of the available resources are being externally supplied to the system, and analogously if one of the $\vec {\hat \varphi } _\sigma ^ \ast$ lies on one of the sides of the simplex, it means that its corresponding species is uptaking and metabolizing only two of the available resources. In general, the positions of $\vec {\hat s}$ and $\vec {\hat \varphi } _\sigma ^ \ast$ depend on the relative ratios with which the resources are supplied or uptaken by the species.

The second condition, which holds in all the scenarios discussed in the previous paragraph, can be interpreted as follows using a graphical representation introduced by Posfai et al. [31] (see Materials and Methods for all the mathematical details). A system with *N*_*R*_ resources can be represented on an (*N*_*R*_ − 1)–dimensional simplex, where each vertex corresponds to one of the available resources; considering for example the case *N*_*R*_ = 3, the system can be represented on a triangle (i.e., a bi-dimensional simplex) as shown in Fig. 4. On this simplex one can draw the vectors $\vec {\hat s}$ and $\vec {\hat \varphi } _\sigma ^ \ast$, whose components are appropriately rescaled versions of (respectively) the resource supply rates *s*_*i*_ and the stationary proteome fractions $\varphi _{\sigma i}^ \ast$ (see Materials and Methods). The second condition for species coexistence prescribes, therefore, that $\vec {\hat s}$ must belong to the *convex hull* of the vectors $\vec {\hat \varphi } _\sigma ^ \ast$, as shown in Fig. 4.

Notice that, differently from similar results of earlier investigations of consumer-resource models [31], this condition involves the *stationary* proteome fractions $\varphi _{\sigma i}^ \ast$, and thus the community has the opportunity to coexist even if the rescaled resource supply rate vector is not within the convex hull of the proteome fractions at the start of the temporal evolution.

Because the CPR model is highly non-linear, it is impossible to predict a priori the values of the stationary fractions $\varphi _{\sigma i}^ \ast$ once all the other parameters are set. However, it is possible to understand how the various parameters affect the dynamics of the system by exploring different regions of the parameter space. The dynamics of the system, in fact, will depend on *how* the proteome fractions *φ*_*σi*_ evolve, and therefore the dynamics of the system will inevitably be influenced by some of the model parameters. In this sense the relevant parameters are the ratios $\gamma _{\sigma i} = \kappa _i^n{\mathrm{/}}\kappa _\sigma ^t$ between the nutritional and translational capacities, and the characteristic timescales *τ*_*σ*_ of the adaptive process that maximizes the growth rate *g*_*σ*_ in the dynamics of *φ*_*σi*_ (see the Materials and Methods for details). The timescales *τ*_*σ*_ measure how fast the dynamics of the proteome fractions *φ*_*σi*_ vary: the smaller *τ*_*σ*_ is, the faster species *σ* can switch between different resources. Biologically speaking, this parameter can be thought of as a measure of how fast the regulatory mechanisms of a microbial species can respond to changes in the availability of resources.

**Fig. 5:**
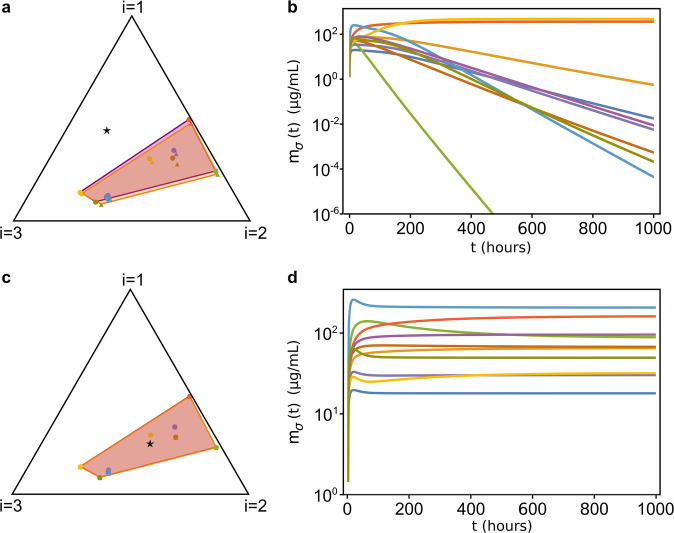
Temporal evolution of the CPR model when $\tau _\sigma \gg 1$ and *γ*_*σi*_ ~ 0.1. **a** Initial conditions for the ${\vec{\varphi}}_\sigma$ of a system with 10 species and 3 resources, depicted using the same graphical representation [31] of Fig. 4: the black triangle is the simplex to which the $\hat \varphi _{\sigma i}$ (colored dots) and the $\hat s_i$ (black star) belong. The initial $\hat \varphi _{\sigma i}$ are represented as colored triangles, and their convex hull is colored in orange, while $\hat \varphi _{\sigma i}^{\ast}$ are represented as circles of the same colors, and their convex hull is in purple. With good approximation, $\hat \varphi _{\sigma i}^ \ast \sim \hat \varphi _{\sigma i}\left( {t = 0} \right)$. **b** Time evolution of the species’ biomasses *m*_*σ*_ relative to the case shown in **a**. Since $\vec {\hat s}$ lies outside of the convex hull of the $\vec {\hat \varphi } _\sigma$, most species go extinct. **c** Same as in **a**, but with $\vec {\hat s}$ belonging to the convex hull of $\vec {\hat \varphi } _\sigma$. **d** Biomass dynamics of the system corresponding to the case shown in **c**. In this case all species coexist. The parameters and the initial conditions were drawn from random distributions (see Supplementary Information). All parameters other than $\vec {\hat s}$ are identical in the four panels).

The first regime that we explored is $\tau _\sigma \gg 1$ and *γ*_*σi*_ ~ 0. In this regime, the adaptive process that regulates the dynamics of the proteome fractions *φ*_*σi*_ is very slow (i.e., species respond very slowly to changes in resource abundance) and the nutritional capacity is much smaller than the translational capacity, which happens for example when species are grown in very low-quality nutrients. In this case, the model predicts that the stationary values $\hat \varphi _{\sigma i}^ \ast$ of the rescaled proteome fractions allocated by the species to nutrient uptake and metabolization change negligibly, and therefore all species survive only if the rescaled nutrient supply rate vector $\vec {\hat s}$ lies in the convex hull of the rescaled *initial* proteome fractions $\vec {\hat \varphi } _\sigma$, as shown in Fig. 5.

**Fig. 6:**
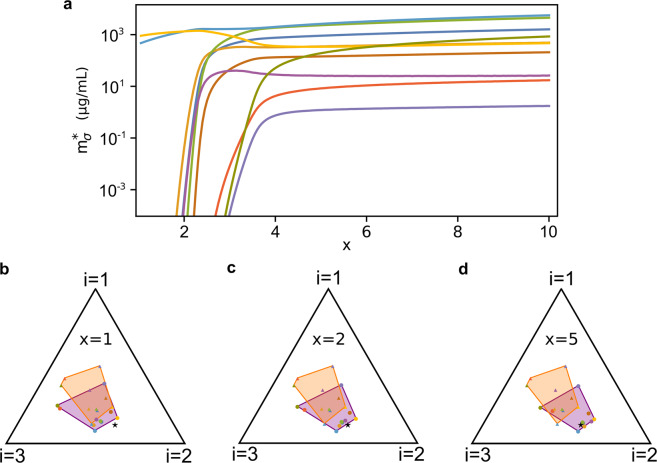
Species coexistence as a function of the rescaled resource supply rate $x{\vec{s}}$ (with *x* > 1). As for Fig. 5, the ${\vec{\varphi}}_\sigma$ evolve according to the CPR model with $\tau _\sigma \gg 1$, $\gamma _{\sigma i} \gtrsim 1$, *N*_*S*_ = 10 and *N*_*R*_ = 3. Here, $\vec {\hat s}$ was drawn randomly outside the convex hull of the initial $\vec {\hat \varphi } _{\sigma}$ (same $\vec{\hat{s}}$ for all panels) and we varied *x* > 1. **a** Stationary values of the species’ biomasses for different values of *x*. When *x* ≃ 1 the system is in an oligodominant phase in which only one or a few species survive, but as *x* grows larger the system shifts to a diverse phase in which all species coexist. Notice that the relative ratios of the stationary abundances $m_\sigma ^ \ast$ are not constant as *x* grows. **b**–**d** Initial (orange) and stationary (purple) convex hull of the rescaled proteome fractions $\hat \varphi _{\sigma i}$ for different values of *x*. For small *x*, the resource supply (black star) is not large enough to allow the $\hat \varphi _{\sigma i}$ to move so that the coexistence condition is satisfied. Increasing *x* (d), this becomes possible and thus all species are able to coexist. The parameters and the initial conditions were drawn from pre-assigned random distributions (see Supplementary Information). All parameters other than $\vec {\hat s}$ and the initial conditions *m*_*σ*_(0) and *c*_*i*_(0) are identical in the four panels.

The second regime we explored is $\tau _\sigma \gg 1$ and $\gamma _{\sigma i} {\gtrsim} 1$. In this case, the dynamics of $\hat \varphi _{\sigma i}$ allows the proteome fractions to move inside the simplex. Therefore, the system can reach stationary states where all species coexist even if $\vec {\hat s}$ is not necessarily close to the convex hull of the initial $\vec {\hat \varphi } _{\sigma}$. On the other hand, we observed that if $\vec {\hat s}$ is too far away from the convex hull of the initial $\vec {\hat \varphi } _{\sigma}$ there might still be extinctions. However, if $\vec {\hat s}$ lies at an intermediate distance between these two cases, the system can reach diverse stationary states only if the resource supply rates *s*_*i*_ are sufficiently large. For example, multiplying each resource supply rate by a factor *x* > 1, i.e. *s*_*i*_ → *xs*_*i*_ (this rescaling leaves $\hat s_i$ unchanged, see Materials and Methods), we observe a transition between two different states of the system for increasing values of *x*: when *x* ~ 1, only a few species survive, whereas for larger values of *x* the stationary biomass densities $m_\sigma ^ \ast$ of the other species increase until all of them coexist. Figure 6 shows an example of such transition. This phenomenon occurs only when $\vec {\hat s}$ lies in specific areas of the simplex, whose shape and position can be determined numerically, but depend on the particular values of the model parameters used. In this same regime, if *γ*_*σi*_ assume increasingly large values (which happens for example, if the species are grown in nutrients with increasingly higher quality) coexistence will be possible even if $\vec {\hat s}$ lies at increasingly large distances from the convex hull of the initial $\vec {\hat \varphi } _{\sigma}$.

**Fig. 7:**
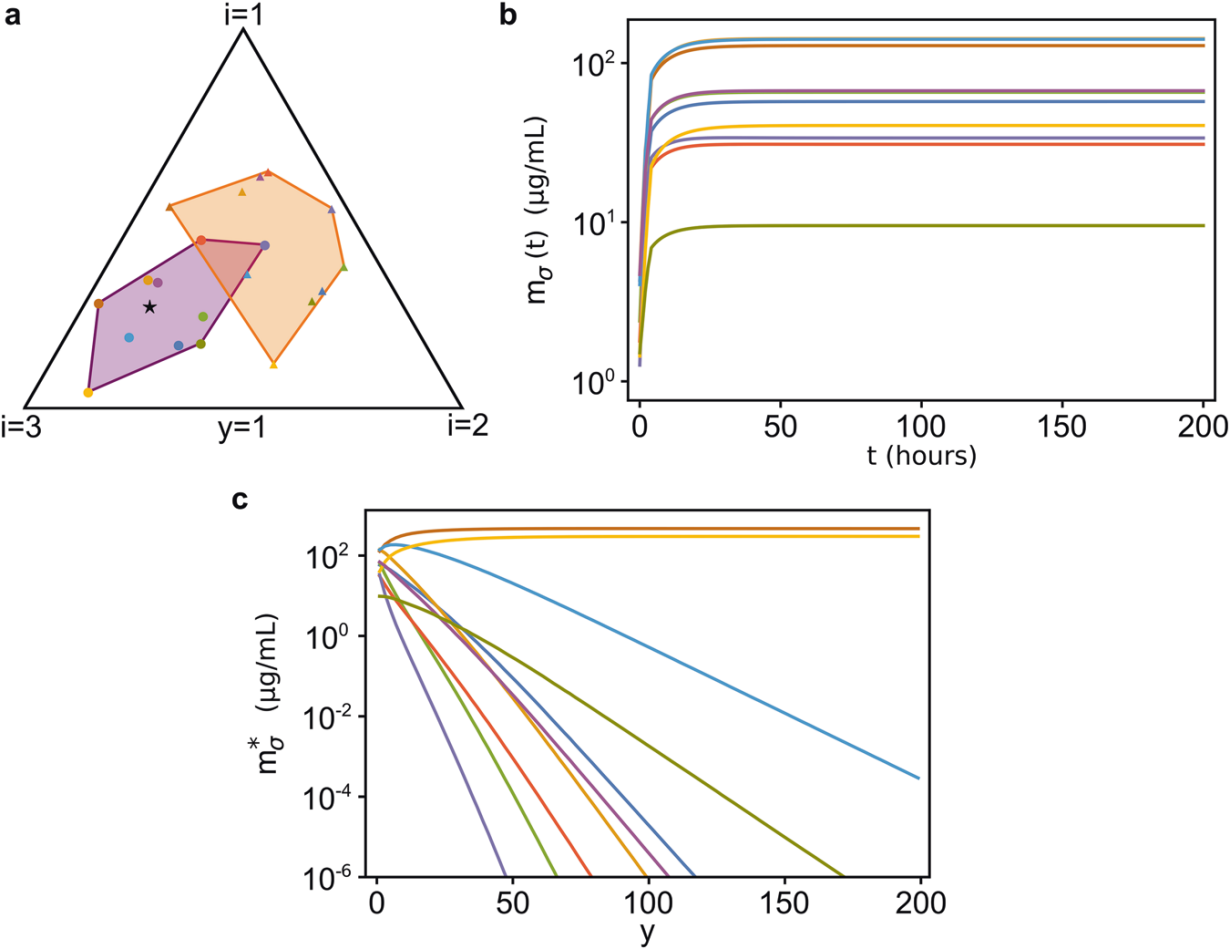
Coexistence in the CPR model is affected by the values of the adaptation timescales *τ*_*σ*_. **a** Initial (orange) and stationary (purple) convex hull of the rescaled proteome fractions $\hat \varphi _{\sigma i}$, for a system with *N*_*S*_ = 10 and *N*_*R*_ = 3 when $\tau _\sigma \,\lesssim\, 1$. **b** Temporal biomass dynamics of the system represented in **a**. **c** Increasing the values of *τ*_*σ*_ leads to extinctions. Shown are the stationary values of species’ biomasses calculated by multiplying the values *τ*_*σ*_ of panels **a**, **b** by a factor *y* > 1, while keeping the other parameters unchanged. As *y* increases, the system shifts from a diverse stationary state for *y* = 1 to states in which only few species survive. The parameters and the initial conditions were drawn from pre-assigned random distributions (see Supplementary Information for more information). All parameters other than *y* are identical in the three panels.

Finally, the last regime we explored is $\tau_\sigma \lesssim 1$, i.e. the adaptive process maximizing species’ growth rates is fast. In this case, the smaller the timescales *τ*_*σ*_ are, the faster the proteome fractions *φ*_*σi*_ will reach their stationary values, and coexistence will always be possible independently of the initial values of the proteome fractions *φ*_*σi*_ and of the resource supply rates *s*_*i*_. However, as the *τ*_*σ*_ grow, fewer and fewer species will be able to coexist. This can be seen by multiplying *τ*_*σ*_ by a factor *y* > 1: Fig. 7 shows how the species’ stationary biomasses change as *y* increases, and we can see that as species adaptation becomes slower (i.e., for larger *y*), fewer and fewer species survive in the community.

The results of this section can be summed up as follows. If metabolic adaptation is slow, i.e. if the characteristic relaxation times *τ*_*σ*_ of the proteome fractions ${\vec{\varphi}}_\sigma$ are large (or in other words, if the species shift slowly between different resources), coexistence will be favored if the system contains high-quality nutrients (i.e., the *γ*_*σi*_ have larger values). If the system contains low-quality nutrients, coexistence will be possible only if the resources are supplied in particular ratios that depend on the species’ proteome allocation. In particular, coexistence will be possible if the rescaled nutrient supply rate vector $\vec {\hat s}$ lies inside the convex hull of the rescaled proteome fractions $\vec {\hat \varphi } _\sigma$. On the other hand, fast metabolic adaptation (i.e., small values of *τ*_*σ*_) always favor coexistence.

## Discussion

Motivated by our experiment that shows how varying proteome allocation can have strong effects on the dynamics of even a very simple microbial community, we have formulated a consumer-resource model that generalizes and incorporates the phenomenological laws discovered by Scott et al. [22]. In this way, we have bridged microbial growth with proteome allocation constraints in competitive communities, and we have investigated the conditions that lead to species coexistence in the presence of multiple resources.

This CPR model describes the population dynamics of a purely competitive microbial community, i.e. an ensemble of species that compete directly for the same pool of resources. The main contribution of this work is introducing a physiological, experimentally-validated constraint on the amount of resources that cells can devote to growth and metabolism in consumer-resource models (i.e., Eq. (15c)). The introduction of this constraint makes it *necessary* to introduce some dynamics on the proteome fractions allocated for nutrient uptake and metabolization, and we have done so using an adaptive approach that assumes that microbial species are evolutionary well adapted to their environment. This work differs (both in scope and approach) from previous ones that involve adaptation on some species’ internal variables [41], and in particular differs from previous works involving the consumer-resource framework [31, 40] that considered phenomenological constraints that were not based on direct experimental measurements, nor on an interpretation of such constraints as arising from the finiteness of the proteome. Introducing the right constraint in such models is particularly important, because the exact conditions that allow species coexistence depend on the specific form of the constraint (see Materials and Methods). A further discussion on the differences between the CPR model and previous ones can be found in the Supplementary Information.

We have then shown that the CPR model predicts that high levels of biodiversity can be achieved only if two conditions apply. The first condition is that the maintenance cost must be proportional to the total proteome fraction allocated by the species to metabolism and growth, i.e. *q*_*σ*_ ∝ Φ_*σ*_. The second condition can be interpreted graphically as described in the Results section, and summarized as follows: (i) if the timescales *τ*_*σ*_ over which the species shift between different resources are large (i.e., $\tau _\sigma \gg 1$) and if the quality of the resources is low, coexistence will be possible only if the resource supply rates have particular values (i.e., the rescaled nutrient supply rate vector $\vec {\hat s}$ belongs to the convex hull of $\vec {\hat \varphi } _\sigma$); (ii) if again $\tau _\sigma \gg 1$, but the resources are of higher quality, coexistence is possible (in some cases the magnitude of the resource supply rates must be large enough), and if the resources’ quality is higher, coexistence is favored; and (iii) coexistence is favored for smaller values of the timescales *τ*_*σ*_. From the biological point of view, these points can be interpreted as follows: (i) if the species switch slowly between different resources and the quality of the resources is low, coexistence will be possible only if the resources are supplied with particular ratios (which depend on the proteome allocation of all the species); (ii) if again the species switch slowly between different resources, coexistence will be favored if the resources have higher quality; (iii) fast metabolic adaptation (i.e., the species can switch quickly between different resources) favors coexistence. Our approach, therefore, makes it possible to quantify precisely in what ways the internal cellular dynamics make coexistence possible in a broad range of environmental contexts.

The dynamics of microbial communities has traditionally been studied at the ecological level by using models of population dynamics describing how the population abundances of different species in the community change over time as the result of competition for resources. While this approach is undoubtedly useful and effective, it often cannot describe the system at a level of detail necessary to make predictions from measurable quantities. In fact, it is becoming increasingly clear that the structure and dynamics of microbial communities are affected by the metabolic activity of the species that comprise them [15–18]. As shown here, mathematical models of community dynamics that take explicitly into account how different species allocate their proteome to regulate nutrient uptake can provide new insights into the link between the ecological properties of microbial communities, i.e. population dynamics and species coexistence, and their intracellular ones, i.e. metabolism and gene expression [20].

Direct competition for resources is only one of the many known interactions that can take place between microbial species: exchange of metabolic byproducts [14], production of toxins [13] and environmental conditioning [42] are only a few of the ways in which we know microbes interact within a community. Each of these processes provide both growth benefits and proteomic costs to microbial species, and can in principle be included in our framework by appropriately taking into account how they affect proteome allocation and species fitness. With our framework it would therefore be possible to make quantitative predictions involving such phenomena, and testing them against experimental data.

## Materials and methods

### The consumer-proteome-resource equations

The derivation of the CPR models equations starts from Eqs. (3a) and (3b). To write these equations explicitly, we introduce the following assumptions: (i) the uptake rate *J*_*σi*_ is proportional to the proteome fraction $\varphi _{\sigma i} = \varphi _{\sigma i}^P$ allocated by species *σ* for the uptake and metabolization of resource *i* and (ii) each resource contributes to the growth of species *σ* through a term $g_\sigma ^{\left( i \right)}$ proportional to the uptake rate *J*_*σi*_, so that the total growth rate *g*_*σ*_ of species *σ* can be written as the sum of all the terms $g_\sigma ^{\left( i \right)}$. Specifically, we rewrite Eq. (1a) as:

9 $$\varphi _{\sigma i}^P = \frac{{\rho _\sigma }}{{\bar \kappa _i^n\left( {c_i} \right)}}g_\sigma ^{\left( i \right)},$$

where *ρ* is considered to be species-dependent, $\bar \kappa _i^n\left( {c_i} \right) = \kappa _i^n \cdot r_i\left( {c_i} \right)$ (with $r_i\left( {c_i} \right) = c_i/\left( {K_i + c_i} \right)$), and $g_\sigma ^{\left( i \right)}$ is the contribution to the growth rate of species *σ* due to the uptake of resource *i*, i.e.:

10 $$g_\sigma = \mathop {\sum }\limits_{i = 1}^{N_R} g_\sigma ^{\left( i \right)},$$

and we generalize Eqs. (1a) and (1b) to:

11a $$g_\sigma = \mathop {\sum }\limits_{i = 1}^{N_R} \frac{{\bar \kappa _i^n\left( {c_i} \right)}}{{\rho _\sigma }}\varphi _{\sigma i}^P,$$

11b $$\varphi _\sigma ^R = \frac{{\rho _\sigma }}{{\kappa _\sigma ^t}}g_\sigma + \varphi _\sigma ^0.$$

Equation (10) implies that the *N*_*R*_ resources are substitutable (e.g., different carbon sources), otherwise, their contribution to the growth rate may satisfy a different equation (e.g., their contributions may be multiplicative rather than additive). We can use Eq. (11a) to write Eq. (11b) in terms of the fractions $\varphi _{\sigma i}$. By doing so we get that the normalization condition given by Eq. (2) reads:

12 $$\mathop {\sum}\limits_{i = 1}^{N_R} {\varphi _{\sigma i}} \left[ {1 + \frac{{\bar \kappa _i^n\left( {c_i} \right)}}{{\kappa _\sigma ^t}}} \right] = 1 - \varphi _\sigma ^Q - \varphi _\sigma ^0: = {\Phi}_\sigma ,$$

where we have written *φ*_*σi*_ instead of $\varphi _{\sigma i}^P$ for simplicity and Φ_*σ*_ is the total proteome fraction that species *σ* allocates to metabolism and biomass synthesis.

We generalize the results of Scott et al. to the case of multiple resources by assuming that the uptake rate *J*_*σi*_ of resource *i* per unit biomass is proportional to *φ*_*σi*_, i.e.:

13 $$J_{\sigma i} = \xi _ir_i\left( {c_i} \right)\varphi _{\sigma i},$$

where the proportionality constant *ξ*_*i*_ can be interpreted biologically as the maximum catalytic rate of the enzyme used to metabolize resource *i* (see Supplementary Information). By comparing Eqs. (13) and (9) we can see that the contribution to the growth rate of species *σ* due to the uptake of resource *i* is proportional to its uptake rate, i.e. $g_\sigma ^{\left( i \right)} = \chi _{\sigma i}J_{\sigma i}$ with

14 $$\chi _{\sigma i}\xi _i = \frac{{\kappa _i^n}}{{\rho _\sigma }}.$$

With the considerations above, we obtain the final equations of the CPR model:

15a $$\dot m_\sigma = m_\sigma \left[ {\mathop {\sum}\limits_{i = 1}^{N_R} {\eta _{\sigma i}r} _i\left( {c_i} \right)\varphi _{\sigma i} - q_\sigma } \right],$$

15b $$\dot c_i = s_i - \xi _ir_i\left( {c_i} \right)\mathop {\sum}\limits_{\sigma = 1}^{N_S} {m_\sigma \varphi _{\sigma i}} ,$$

15c $$\mathop {\sum}\limits_{i = 1}^{N_R} {\varphi _{\sigma i}} \left[ {1 + \gamma _{\sigma i}r_i\left( {c_i} \right)} \right] = {\Phi}_\sigma ,$$

where we have written explicitly $\bar \kappa _i^n\left( {c_i} \right) = \kappa _i^nr_i\left( {c_i} \right)$ with $r_i\left( {c_i} \right) = c_i/\left( {K_i + c_i} \right)$, and we have defined $\eta _{\sigma i}: = \kappa _i^n/\rho _\sigma$ and $\gamma _{\sigma i}: = \kappa _i^n/\kappa _\sigma ^t$ to simplify the notation. Regardless of the particular form of *r*(*c*) chosen, for our purposes we only need to assume that *r*(*c*) is a monotonically increasing function of *c*, and that ${\mathrm{lim}}_{c \to 0}r\left( c \right)/c = 1/K$ and ${\mathrm{lim}}_{c \to \infty }r\left( c \right) = 1$.

The constraint in Eq. (15c) is the explicit expression of Eq. (2) in our framework, and can be interpreted geometrically: considering species *σ*, the *N*_*R*_-dimensional vector ${\vec{\varphi}}_\sigma = \left( {\varphi _{\sigma 1}, \ldots ,\varphi _{\sigma N_R}} \right)$ belongs to a hyperplane whose normal vector $\hat n_\sigma$ has components $1 + \gamma _{\sigma i}r_i\left( {c_i} \right)$. This means that as the system evolves, the components of $\hat n_\sigma$ vary with time and therefore the hyperplane to which ${\vec{\varphi}}_\sigma$ belongs moves in the *N*_*R*_-dimensional space. This is also the reason why the proteome fractions *φ*_*σi*_ must be dynamical variables: the coefficients 1 + *γ*_*σi*_*r*_*i*_(*c*_*i*_) in Eq. (15c) are not fixed, but change with time depending on the system’s dynamics through *r*_*i*_(*c*_*i*_). This implies that for the constraint to be satisfied at all times, the proteome fractions *φ*_*σi*_ *cannot* be fixed but must be, in turn, dynamical variables: an increase (decrease) of 1 + *γ*_*σi*_*r*_*i*_(*c*_*i*_) must be balanced by a decrease (increase) of some of the *φ*_*σi*_. This constraint reflects the well known fact that microbes can vary their enzyme synthesis with time and switch between nutrients according to environmental conditions [40, 43–45].

### Dynamics of the proteome fractions *φ*_*σi*_

We call ${\vec{c}} = \left( {c_1, \ldots ,c_{N_R}} \right)$ the vector of resource concentrations and define

16 $$F_\sigma \left( {{\vec{\varphi}}_\sigma ,{\vec{c}}} \right): = \mathop {\sum}\limits_{i = 1}^{N_R} {\varphi _{\sigma i}} \left[ {1 + \gamma _{\sigma i}r_i\left( {c_i} \right)} \right] - {\Phi}_\sigma$$

so that the constraint given by Eq. (15c) can be written more simply as $F_\sigma \left( {{\vec{\varphi}}_\sigma ,{\vec{c}}} \right) = 0$. Since this constraint must hold at every instant, any equation for ${\vec{\varphi}}_\sigma$ must satisfy

17 $$\dot F_\sigma \left( {\vec{\varphi}}_\sigma ,{\vec{c}} \right) \equiv \dot {\vec{\varphi}}_\sigma \cdot {\vec{\nabla}}_\varphi F_\sigma + \dot {\vec{c}} \cdot {\vec{\nabla}}_cF_\sigma = 0,$$

where ${\vec{\nabla}}_\varphi$ and ${\vec{\nabla}}_c$ are, respectively, the gradients taken with respect to the components of ${\vec{\varphi}}_\sigma$ and ${\vec{c}}$. The “minimal” equation for *φ*_*σi*_, i.e. the simplest one (in the sense that it does not introduce extra terms orthogonal to ${\vec{\nabla}}_\varphi F_\sigma$, which would lead to a proliferation of new parameters) that satisfies Eq. (17) is therefore:

18 $$\dot {\vec{\varphi}}_\sigma = - \frac{{\vec{\nabla} _\varphi F_\sigma }}{{\left( {\vec{\nabla}_\varphi F_\sigma } \right)^2}}\vec{c} \cdot \vec{\nabla}_cF_\sigma ,$$

where, however, we are not taking into account the fact that with such an equation some of the *φ*_*σi*_ might become negative with time (see Supplementary Information for detailed computations on how this can be taken into account).

Microbes are able to switch between nutrients when cultured in mediums containing more than one resource [43]. For this reason, we can implement an adaptive approach [40] and ask that ${\vec{\varphi}}_\sigma$ evolves in time so that the growth rate *g*_*σ*_ of species *σ* is maximized respecting the constraint $F_\sigma \left( {{\vec{\varphi}}_\sigma ,\vec{c}} \right) = 0$, i.e. Equation (15c) is satisfied. In this case the evolution equation for ${\vec{\varphi}}_\sigma$ becomes:

19 $$\dot {\vec{\varphi}}_\sigma = \frac{1}{{\tau _\sigma }}\vec{\nabla}_\varphi g_\sigma - \frac{{\vec{\nabla}_\varphi F_\sigma }}{{\left( {\vec{\nabla}_\varphi F_\sigma } \right)^2}}\left( {\frac{1}{{\tau _\sigma }}\vec{\nabla} _\varphi g_\sigma \cdot \vec{\nabla}_\varphi F_\sigma + \dot{\vec{c}} \cdot \vec{\nabla}_cF_\sigma } \right),$$

where we have introduced *τ*_*σ*_, the characteristic timescale over which ${\vec{\varphi}}_\sigma$ changes [40] (detailed computations are shown). We can recover Eq. (18) from Eq. (19) by sending *τ*_*σ*_ to infinity. Geometrically, Eq. (18) represents the case in which ${\vec{\varphi}}_\sigma$ is dragged along by the hyperplane to which it belongs, as the hyperplane moves because of Eq. (15c). On the other hand, according to Eq. (19) (with small enough values of *τ*_*σ*_) the ${\vec{\varphi}}_\sigma$ are free to move on the hyperplane to find the maximum instantaneous growth rate compatible with the constraint given by Eq. (15c).

In this work we have used a generalization of Eq. (19) that ensures $\varphi _{\sigma i}\left( t \right) \ge 0\forall t$, and varied the values of *τ*_*σ*_ when needed (see Supplementary Information for details).

The introduction of this dynamics on the proteome fractions *φ*_*σi*_ in consumer-resource models allows our model to reproduce phenomena that classic consumer-resource theory cannot describe, like diauxic shifts (see Fig. S.2).

### Conditions for coexistence

Evaluating Eqs. (15a)–(15c) at stationarity we obtain:

20a $$\mathop {\sum}\limits_{i = 1}^{N_R} {\eta _{\sigma i}r_i^ \ast \varphi _{\sigma i}^ \ast } = q_\sigma ,$$

20b $$s_i = \xi _ir_i^ \ast \mathop {\sum}\limits_{\sigma = 1}^{N_S} {m_\sigma ^ \ast \varphi _{\sigma i}^ \ast } ,$$

20c $$\mathop {\sum}\limits_{i = 1}^{N_R} {\varphi _{\sigma i}^ \ast } \left( {1 + \gamma _{\sigma i}r_i^ \ast } \right) = {\Phi}_\sigma ,$$

where we are denoting with the symbol “*” the quantities computed at stationarity, and we have assumed *m*_*σ*_ ≠ 0. If we now assume $\varphi _{\sigma i}^ \ast \,\ne\, 0$ for all *i* and all species, it is easily seen by substitution that a possible solution for $r_i^ \ast$ in Eqs. (20a) and (20c) is

21 $$r_i^ \ast = \left[ {\kappa _i^n\left( {\frac{{{\Phi}_\sigma }}{{\rho _\sigma q_\sigma }} - \frac{1}{{\kappa _\sigma ^t}}} \right)} \right]^{ - 1}.$$

Under our assumption (i.e., $\varphi _{\sigma i}^ \ast \,\ne\, 0$ for all *i*, for all species), and if *N*_*S*_ > *N*_*R*_ (i.e., the number of species is larger than the number of resources) this solution is acceptable only if its right-hand side is independent of *σ*, i.e. if

22 $$\frac{{{\Phi}_\sigma }}{{\rho _\sigma q_\sigma }} - \frac{1}{{\kappa _\sigma ^t}} = \Theta ,$$

with Θ some given constant independent of *σ*. Using Eqs. (21) and (22) in Eqs. (20c) or (20a) we get

23 $$\mathop {\sum}\limits_{i = 1}^{N_R} {\varphi _{\sigma i}^ \ast } = \frac{{{\Phi}_\sigma }}{{1 + \frac{1}{{\Theta \kappa _\sigma ^t}}}}.$$

From Eq. (21) we have:

24 $$r_i^ \ast = \frac{1}{{\kappa _i^n\Theta }} \quad \Rightarrow \quad c_i^ \ast = \frac{{K_i}}{{\kappa _i^n\Theta - 1}},$$

and since we need $r_i^ \ast \,<\, 1$ (or equivalently $c_i^ \ast \,> \, 0$), we need $\Theta \,> \, {\mathrm{max}}_i1/\kappa _i^n$. Therefore, Eq. (22) can be rewritten as

25 $$q_\sigma = \frac{{{\Phi}_\sigma }}{{\rho _\sigma \left( {\Theta + 1/\kappa _\sigma ^t} \right)}},$$

which is the explicit expression of the relationship between *q*_*σ*_ and Φ_*σ*_. Equation (23) is a consequence of the system’s constraint in Eq. (20c), which is Eq. (15c) computed at stationarity. Therefore, the expression of the maintenance cost given in Eq. (25) is a consequence of the constraint introduced in the CPR model.

Notice, again, that this holds under the assumption that $\varphi _{\sigma i}^{\ast} \,\ne\, 0$ for all *i* and *σ,* and *N*_*S*_ > *N*_*R*_. If we remove these assumptions, then it is possible to find solutions with *N*_*S*_ ≤ *N*_*R*_ where Eq. (22) does not hold. For example, if the species’ stationary proteome fractions ${\vec{\varphi}}_\sigma ^ \ast$ are non-overlapping (i.e., ${\vec{\varphi}}_\sigma ^ \ast \cdot {\vec{\varphi}}_\rho ^ \ast = 0$ when *σ* ≠ *ρ*), then $r_i^ \ast$ as given in Eq. (21) can be a valid solution without requiring Eq. (22). Consider as an example the particular case *N*_*S*_ = *N*_*R*_ = 3 and $\varphi _{\sigma i}^ \ast \propto \delta _{\sigma i}$ (where *δ* is Kronecker’s delta), i.e. a system with three species where each one uptakes only one resource, and no two species uptake the same resource. It is easy to imagine that the three species should be able to coexist, since their niches (defined in this context as the set of resources used for sustenance) do not overlap. This is indeed the case, given that a solution for $r_i^ \ast$ in Eqs. (20a) and (20c) is given by:

26 $$r_i^ \ast = \left[ {\kappa _i^n\left( {\frac{{{\Phi}_i}}{{\rho _iq_i}} - \frac{1}{{\kappa _i^t}}} \right)} \right]^{ - 1},$$

where we have identified each species index *σ* with the only resource *i* it consumes, and we don’t need to require Eq. (22) to hold for this solution to be feasible. This will be of course true even for systems where the species and/or resource labels are permutated (e.g., species 1 uptakes resource 2, species 2 uptakes resource 3 and species 3 uptakes resource 1, instead of species 1 uptaking resource 1, species 2 uptaking resource 2, and species 3 uptaking resource 3). This will be true even when *N*_*R*_ > *N*_*S*_, as long as the vectors ${\vec{\varphi}}_\sigma ^ \ast$ are still non-overlapping and the inverse of *r*_*i*_ is written as the product of $\kappa _i^n$ and ${\Phi}_\sigma /\left( {\rho _\sigma q_\sigma } \right) - 1/\kappa _\sigma ^t$ where *σ* is the (only) species uptaking that resource. If one of the resources, e.g. resource *j*, is not uptaken by any species one has $\dot c_j = s_j$, i.e. *c*_*j*_ will grow linearly indefinitely. On the other hand, if *N*_*S*_ > *N*_*R*_ then Eq. (22) is necessary in order to have feasible solutions, even if we remove the assumption that $\varphi _{\sigma i}^{\ast} \,=\, 0$ for all species and resources.

Going back to Eqs. (20a)–(20c), if we now define:

27a $$\hat s_i = \frac{{s_i\kappa _i^n{\mathrm{/}}\xi _i}}{{\mathop {\sum}\nolimits_{j = 1}^{N_R} {s_j\kappa _j^n} {\mathrm{/}}\xi _j}}$$

27b $$\hat \varphi _{\sigma i}^ \ast = \frac{{\varphi _{\sigma i}^ \ast }}{{\mathop {\sum}\nolimits_{j = 1}^{N_R} {\varphi _{\sigma j}^ \ast } }},$$

27c $$z_\sigma = \frac{{m_\sigma ^ \ast \rho _\sigma q_\sigma }}{{\mathop {\sum}\nolimits_\lambda {m_\lambda ^ \ast } \rho _\lambda q_\lambda }},$$

(so that *z*_*σ*_ are positive coefficients that sum to one), and Eq. (20b) can be rewritten as

28 $$\hat s_i = \mathop {\sum}\limits_{\sigma = 1}^{N_S} {z_\sigma \hat \varphi _{\sigma i}}$$

(see Supplementary Information for the detailed computations). Since $\mathop {\sum}\nolimits_i {\hat s_i} = \mathop {\sum}\nolimits_i {\hat \varphi _{\sigma i}^ \ast } = 1$, the vectors $\vec {\hat s}$ and $\vec {\hat \varphi } _\sigma ^ \ast$ belong to an (*N*_*R*_ − 1)–dimensional simplex. Furthermore, since *z*_*σ*_ are positive coefficients that sum to one, Eq. (28) means that $\vec {\hat s}$ belongs to the convex hull of the vectors $\vec {\hat \varphi } _\sigma ^ \ast$. Since Eq. (28) derives from requiring that all species have non-null stationary biomasses, we can see how this is the other condition necessary for coexistence.

At first glance, the result in Eq. (28) looks similar to what has been observed in consumer-resource model with metabolic trade-offs by Posfai et al. [31]. However, our result has an important difference with respect to that model: Eq. (28) depends in fact on the (rescaled) value of *φ*_*σi*_ *at stationarity*. In the CPR model, therefore, the proteome fractions *φ*_*σi*_ vary over time to satisfy Eq. (28), i.e. to include $\vec {\hat s}$ in the convex hull of the vectors $\vec {\hat \varphi } _\sigma ^ \ast$, unlike in Posfai et al. [31] where metabolic strategies (which in our framework correspond to the *φ*_*σi*_) are fixed and thus coexistence is only possible if $\vec {\hat s}$ is within the convex hull of the *φ*_*σi*_ from the very start.

If we now suppose that $\tau _\sigma \gg 1$, so that we can use Eq. (18) for the dynamics of *φ*_*σi*_, observing that the *i*-th component of the gradients $\vec{\nabla}_\varphi F_\sigma$ and $\vec{\nabla}_cF_\sigma$ are

29a $$\left( {\vec{\nabla}_\varphi F_\sigma } \right)_i \equiv \frac{{\partial F_\sigma }}{{\partial \varphi _{\sigma i}}} = 1 + \gamma _{\sigma i}r_i\left( {c_i} \right)$$

and

29b $$\left( {\vec{\nabla}_cF_\sigma } \right)_i \equiv \frac{{\partial F_\sigma }}{{\partial c_i}} \propto \gamma _{\sigma i},$$

we find that if *γ*_*σi*_ ~ 0 then $\dot {\vec{\varphi}}_\sigma \sim 0$ and therefore $\varphi _{\sigma i}^ \ast \sim \varphi _{\sigma i}\left( {t = 0} \right)$. In other words, if the *γ*_*σi*_ are small, the proteome fractions *φ*_*σi*_ at stationarity will be close to their initial values. Therefore in this case, with good approximation, Eq. (28) gives the condition for all species to coexist, i.e. $\vec {\hat s}$ must be inside the convex hull of $\hat \varphi _{\sigma i} = \varphi _{\sigma i}\left( 0 \right)/\mathop {\sum}\nolimits_j {\varphi _{\sigma j}} \left( 0 \right)$. If $\gamma_{\sigma i}\gtrsim1$ as discussed in the Results section, on the other hand, coexistence will be possible if the components of $\dot {\vec{\varphi}}_\sigma$ are not too small for a sufficiently long period of time so as to allow them to reach values satisfying Eq. (28) and thus for the species to coexist. This can be obtained by using large supply rates *s*_*i*_ so that *r*_*i*_(*c*_*i*_) ~ 1 for a sufficiently long time, as discussed in the Results. Finally, if the ratios *γ*_*σi*_ have larger values the proteome fractions *φ*_*σi*_ will be able to move more quickly.

### Strains used in the experiment

The *Escherichia coli* strains used in our experiment have the same genetic background MG1655. The strains used in the experiments were constructed starting from the ancestor strain 0Y (expressing constitutively the yellow fluorescent protein mVenus from the genome, with genotype attTN7::pRNA1_mVenus) or the ancestor strain 0R (expressing constitutively the red fluorescent protein mKate2Hyb from the genome, with genotype attTN7::pRpsL_mKate2Hyb).

Strain 1 was obtained by transforming strain 0Y with the plasmid pR (see Table S.1), which contains the ampicillin resistance cassette, the red fluorescent protein mCherry under the control of the *trc* promoter, a hybrid of the *trp* and *lac* promoters, and the *lac* repressor, *lacI*. The expression of mCherry could thus be induced by adding IPTG, which binds to the repressor encoded by *lacI* allowing the expression of genes promoted by the *trc* promoter (here, mCherry). Because IPTG cannot be metabolized by *E. coli*, its concentration remains constant during our experiment and is unaltered by bacterial growth.

Strain 2 was obtained by transforming strain 0R with the plasmid pAMP (see Table S.1), which was obtained by removing the inducible red fluorescent protein mCherry from plasmid pR using traditional cloning.

Strain 3 was obtained by transforming strain 0R with plasmid pY (see Table S.1), which is identical to plasmid pR, except for the fluorescent protein induced by the *trc* promoter, which is Venus YFP instead of mCherry.

Strain 4 was obtained transforming strain 0Y with plasmid pAMP.

Because all strains had the ampicillin resistance cassette in the plasmids used to transform them, we performed the experiments by adding ampicillin to the medium to prevent contamination and plasmid loss.

Figures S.3–S.14 show the results of fitness assays performed with all the strains used in our experiments and the ancestor strains.

### Experimental protocol

The competition assays were performed as follows:

1. The strains were cultured overnight from a stock culture in M63 medium with 1% w/v glucose, and ampicillin. Then, the strains were mixed to perform competition assays aiming for 50:50 relative frequencies.
2. The mixtures were inoculated in a 96-well plate containing M63 medium with 1% w/v glucose and ampicillin at eight different IPTG concentrations: 0, 15, 30, 45, 60, 75, 90, 105 μM (six technical replicates per concentration).
3. The well plate was covered with a porous rayon film that allowed gas exchange and was cultured for 24 h at 30 °C on a microplate shaker set at 1050 rpm.
4. After 24 h, the plate was reinoculated in a new 96-well plate with fresh medium (with the appropriate concentrations of IPTG in each well) with a dilution factor of 100. The new plate was cultured for another cycle at 30 °C for 24 h with constant shaking at 1050 rpm, while the old one was diluted with a dilution factor of 2000 to be analyzed at the flow cytometer.

### IPTG calibration and computation of the normalized protein production rate

We measured how the fluorescence intensity of individual cells, a proxy for the total amount of fluorescent protein produced, varied as a function of the IPTG concentration. To do so, we inoculated strains 1 and 3 in a 96-well plate containing M63 minimal medium with ampicillin, 1% w/v glucose and the same IPTG concentrations used in our experimental protocol (six technical replicates per concentration, per strain). The plate was incubated at 30 °C for 8 h with constant shaking at 1050 rpm. At times *t* = 4 h and *t* = 8 h after inoculation we measured at the flow cytometer the mean fluorescence intensity of cells due to the induced fluorescent proteins at the various concentrations of IPTG (Fig. 3d, e). From these data, we estimated the normalized fluorescent protein production rate as follows.

We call *k*(*C*_*I*_) the rate at which the fluorescence of the inducible protein increases when cells are exposed to a concentration *C*_*I*_ of IPTG, and we call *d*_*FP*_ the fluorescent protein degradation rate. The fluorescent intensity *I* of a cell (due to the production of the IPTG-inducible fluorescent protein) in between two successive cell divisions thus satisfies $dI/dt = k\left( {C_I} \right) - d_{FP}I$. At a cell division event, the fluorescent intensity of a cell is reduced by a factor 2. Indicating with *I*_0_ the cell’s fluorescent intensity at the first measurement time (*t* = 4 h), it can be shown (see Supplementary Information) that according to this model the cell’s fluorescent intensity changes with time as:

30 $$I\left( t \right) =	 2I_0e^{ - gt\left( {1 + \frac{{d_{FP}}}{g}} \right)} + \frac{{k\left( {C_I} \right)}}{{d_{FP}}}\left[ {1 - e^{ - gt\left( {1 + \frac{{d_{FP}}}{g}} \right)}} \right]\\ 	\left( {1 - \frac{1}{{2^{1 + \frac{{d_{FP}}}{g}} - 1}}} \right),$$

where *g* is the cell’s growth rate. Fluorescent proteins have small degradation rates compared to the cellular growth rate, so assuming $d_{FP} \ll g$ we can approximate Eq. (30) as:

31 $$I\left( t \right) = 2I_0e^{ - gt} + 2{\mathrm{ln}}\left( 2 \right)\frac{{k\left( {C_I} \right)}}{g}\left( {1 - e^{ - gt}} \right).$$

We used Eq. (31) and the data in Fig. 3d, e to compute the quantity *k*(*C*_*I*_). Because the absolute value of *k*(*C*_*I*_) depends on the arbitrary units returned by the flow cytometer (the intensity *I* is measured as a cell’s pulse area at the flow cytometer), we normalized the values of *k*(*C*_*I*_) dividing them by the mean fluorescent intensity 〈*I*〉 of cells measured in the absence of IPTG at the first measurement in the calibration experiment (see Fig. 3d, e). Such a normalization affects only the absolute value of such rates, and not their relative magnitude. This also means that the normalized production rates shown for the two experiments in Fig. 3 cannot be compared directly.

The normalized *k*(*C*_*I*_)/〈*I*〉 are the protein production rates of strains 1 and 3 (with dimensions 1/time) reported in Fig. 3.

The growth curves and the growth rates of strains 1 and 3 for the different IPTG concentrations used in our experiments are shown in Figs. S.15–S.18.

### Estimation of the selection coefficient *S*

To first approximation, we can use the results of Scott et al. [22] on the dependence of the exponential growth rate of *E. coli* strains grown in isolation in rich medium (which in our notation corresponds to *r*(*c*) = 1) to estimate the outcome of our competition experiment, and in particular to estimate the dependence of the selection coefficient on the Φ_*σ*_. From Eq. (11b) the growth rate of species *σ* is given by:

32 $$g_\sigma = \frac{{\kappa _\sigma ^t}}{{\rho _\sigma }}\left( {\varphi _\sigma ^R - \varphi _\sigma ^0} \right).$$

Using also Eq. (11a) with *N*_*R*_ = 1 and the definition of Φ_*σ*_ from Eq. (12), we can rewrite this as:

33 $$g_\sigma = \frac{{\kappa _\sigma ^t}}{{\rho _\sigma }}\left( {{\Phi}_\sigma - \frac{{\rho _\sigma }}{{\kappa ^n}}g_\sigma } \right),$$

which is easily rearranged into:

34 $$g_\sigma = \frac{{\kappa _\sigma ^t\kappa ^n}}{{\rho _\sigma \left( {\kappa _\sigma ^t + \kappa ^n} \right)}}{\Phi}_\sigma$$

(see also Eq. (S23) in [22, Online Supporting Material]). Therefore, if we assume $\kappa _1^t = \kappa _2^t$ and *ρ*_1_ = *ρ*_2_ (which can happen, for example, if the two populations are different strains of the same microbial species with similar genetic backgrounds) and $m_\sigma \left( t \right) = m_\sigma \left( 0 \right){\mathrm{exp}}\left( {g_\sigma t} \right)$ (which is a good approximation for populations growing in batch cultures with nutrient-rich medium), the selection coefficient is given by:

35 $$S = \frac{d}{{dt}}{\mathrm{ln}}\frac{f}{{1 - f}} = \frac{{\dot m_1}}{{m_1}} - \frac{{\dot m_2}}{{m_2}} = \frac{{\kappa ^t\kappa ^n}}{{\rho \left( {\kappa ^t + \kappa ^n} \right)}}\left( {{\Phi}_1 - {\Phi}_2} \right),$$

where *f* = *m*_1_/(*m*_1_ + *m*_2_) and 1 − *f* = *m*_2_/(*m*_1 _+ *m*_2_) are the relative abundances (or “frequencies”) of strain 1 and strain 2, respectively. The time series of the values of ln(*f*/1 − *f*) for the two experiments are shown in Figs. S.19 and S.20.

If we now apply the CPR model, i.e. Eqs. (15a)–(15c), to the case of two populations and one resource, we obtain:

36a $$\dot m_\sigma = m_\sigma \left( {\eta _\sigma r\left( c \right)\varphi _\sigma - q_\sigma } \right) \qquad \quad \sigma = 1,2,$$

36b $$\dot c = s - \xi r\left( c \right)\left( {m_1\varphi _1 + m_2\varphi _2} \right),$$

36c $$\varphi _\sigma \left( {1 + \gamma _\sigma r\left( c \right)} \right) = {\Phi}_\sigma \qquad \quad \sigma = 1,2,$$

where $\eta _\sigma = \kappa ^n{\mathrm{/}}\rho _\sigma$, $\gamma _\sigma = \kappa ^n{\mathrm{/}}\kappa _\sigma ^t$, and now Eq. (36c) gives the explicit expression of the (only) proteome fraction *φ*_*σ*_ as a function of the resource concentration. Because the ancestors of our two strains (i.e., strains 0Y and 0R) have the same genetic background (see, for example, Figs. S.3 and S.4), we set *η*_1_ = *η*_2_ = *η*, *q*_1_ = *q*_2_ = *q* and *γ*_1_ = *γ*_2_ = *γ* in Eqs. (36a)–(36c). Notice that, instead, Φ_1_ ≠ Φ_2_ because the proteome allocation of strain 1 could be varied experimentally and because the plasmids introduced in the ancestor strains have different maintenance costs. Furthermore, note that assuming *η*_1_ = *η*_2_ is equivalent to assuming *ρ*_1_ = *ρ*_2_, and on the other hand *γ*_1_ = *γ*_2_ is equivalent to $\kappa _1^t = \kappa _2^t$. Given that cells in the experiment are grown in nutrient-rich conditions, we assume that the maintenance cost is negligible, i.e. *q* ≃ 0. Furthermore, because most of the dynamics (i.e., the relative change in abundance of the two strains) occurs in the early phases of growth when glucose is abundant, we assume that *r*(*c*) ≈ 1 at all times so that we can neglect Eq. (36b) and we are left with:

37 $$\dot m_\sigma = m_\sigma \frac{\eta }{{1 + \gamma }}{\Phi}_\sigma \sigma = 1,2.$$

Notice again that this expression, and in particular the fact that the growth rate of species *σ* is proportional to Φ_*σ*_, is a consequence of the constraint in Eq. (36c). We, therefore, have that the expression of the selective advantage *S* in this case is:

38 $$S = \frac{d}{{dt}}{\mathrm{ln}}\frac{f}{{1 - f}} = \frac{{\dot m_1}}{{m_1}} - \frac{{\dot m_2}}{{m_2}} = \frac{\eta }{{1 + \gamma }}\left( {{\Phi}_1 - {\Phi}_2} \right).$$

From the definitions of *η* = *κ*^*n*^/*ρ* and *γ* = *κ*^*n*^/*κ*^*t*^ it is immediate to see that the coefficient in Eq. (38) is the same as the one in Eq. (35).

With our framework, however, we can show that this result continues to be true even when we remove the assumption that *r*(*c*) = 1 at all times. In our experiment, for example, it was not true that glucose was always abundant throughout the experiment, since the density of the cells saturated well before the following re-inoculation in fresh medium was made (i.e., 24 h). In fact, the typical growth rate of the strains, estimated from growth curves measured in the same experimental conditions used for the competition assays, is 0.3 1/h. The competition assays started from a cellular density of ~8 · 10^6^ cells/mL, thus if growth was exponential the density after 24 h would have been be ~1.4 · 10^10^, which is much higher than the typical density (~10^9^ cells/mL) that *E. coli* cells reach at saturation. With a growth rate of 0.3 1/h, the time needed to reach a cellular density that is hundredfold the initial one (and therefore the time needed to reach saturation after a re-inoculation) is ~15.4 h.

A model better suited to describe the population dynamics of the two strains in our experiment would be as follows. The temporal dynamics of biomass and glucose concentration between two consecutive dilutions satisfies:

39a $$\dot m_\sigma = m_\sigma \eta _\sigma r\left( c \right)\varphi _\sigma \qquad \quad \sigma = 1,2$$

39b $$\dot c = - r\left( c \right)\left( {m_1\eta _1\varphi _1 + m_2\eta _2\varphi _2} \right),$$

where *c*(*t*) is the concentration of glucose at time *t* and *r*(*c*) = *c*/(*c* + *K*) is Monod’s function. This model is somewhat similar to a classic consumer-resource model, with the difference that there is no mortality term in Eq. (39a): between two consecutive dilutions, the biomass *m*_*σ*_ of strain *σ* will grow as long as there is glucose available, and because *r*(0) = 0 the strains will stop growing (i.e. they will enter the stationary phase) once glucose runs out.

We now make the following approximation: we assume that, after every reinoculation, glucose is initially abundant (i.e. *r* ~ 1) and that the transition of *r*(*c*) from 1 to 0 as *c* decreases is abrupt, which happens if *K* is sufficiently small. In other words, we assume that *K* is sufficiently small so that *r*(*c*) ≈ 1 until a given time *T* (the instant at which glucose is completely depleted), when *r*(*c*) abruptly goes to zero (i.e., *r*(*c*(*t*)) ~ *H*(*T* − *t*) with *H* the Heaviside’s step function). This means that after a reinoculation *m*_*σ*_ will grow exponentially for a time interval of length *T*, after which it will stop until the next dilution. If we still set *η*_1_ = *η*_2_ = *η* and *γ*_1_ = *γ*_2_ = *γ* and call *D* the dilution factor between reinoculations, we have that the biomass $m_\sigma ^{\left( N \right)}$ of strain *σ* at the *N*-th dilution is ($m_\sigma ^{\left( 0 \right)}$ being the biomass at the initial inoculation):

40a $$m_\sigma ^{\left( 1 \right)} = m_\sigma ^{\left( 0 \right)}{\mathrm{exp}}\left( {\frac{\eta }{{1 + \gamma }}{\Phi}_\sigma T} \right),$$

40b $$m_\sigma ^{\left( 2 \right)} = \frac{{m_\sigma ^{\left( 1 \right)}}}{D}{\mathrm{exp}}\left( {\frac{\eta }{{1 + \gamma }}{\Phi}_\sigma T} \right) = \frac{{m_\sigma ^{\left( 0 \right)}}}{D}{\mathrm{exp}}\left( {2\frac{\eta }{{1 + \gamma }}{\Phi}_\sigma T} \right),$$

40c $$m_\sigma ^{\left( 3 \right)} =	 \frac{{m_\sigma ^{\left( 2 \right)}}}{D}{\mathrm{exp}}\left( {\frac{\eta }{{1 + \gamma }}{\Phi}_\sigma T} \right) = \frac{{m_\sigma ^{\left( 0 \right)}}}{D}{\mathrm{exp}}\left( {3\frac{\eta }{{1 + \gamma }}{\Phi}_\sigma T} \right),$$

$$\hskip 110pt\vdots$$

40d $$m_\sigma ^{\left( N \right)} = \frac{{m_\sigma ^{\left( 0 \right)}}}{{D^{N - 1}}}{\mathrm{exp}}\left( {N\frac{\eta }{{1 + \gamma }}{\Phi}_\sigma T} \right).$$

Therefore, if we call *f*^(*N*)^ the relative abundance of strain 1 at the *N*-th dilution, we have:

41 $${\mathrm{ln}}\frac{{f^{\left( N \right)}}}{{\left( {1 - f} \right)^{\left( N \right)}}} = {\mathrm{ln}}\frac{{m_1^{\left( 0 \right)}}}{{m_2^{\left( 0 \right)}}} + NT\frac{\eta }{{1 + \gamma }}\left( {{\Phi}_1 - {\Phi}_2} \right),$$

which gives the same expression for the selection coefficients after deriving with respect to the time *NT*.

### Comments on the experimental selection coefficient *S*

Figure 3a shows that strain 1 has a fitness advantage over strain 2 in the absence of IPTG, since *S* > 0 at low protein production rates, even though the only significant difference between the two strains is that strain 1 carries an extra copy of *lacI* and the inducible fluorescent protein mCherry (see “Strains used in the experiment”); in our theoretical framework, such an advantage implies that ${\Phi}_1^{\left( 0 \right)} - {\Phi}_2 \,> \, 0$. This may be explained by the observation that expressing *lacI* is beneficial for *E. coli* strains growing on glucose because it represses expression of the *lac* operon. Stoebel et al. [46] have in fact found that cells with the genomic copy of *lacI* show some residual *lacA* activity when grown in glucose, and estimated the cost of expressing *lacA* as 1.85% per generation [46], which may be alleviated in the presence of an extra copy of *lacI*. See also Supplementary Information for a more detailed discussion. Using our data, it is possible to estimate the ratio Φ_1_/Φ_2_ at different protein production rates (Fig. 3c). This ratio is approximately ${\Phi}_1^{\left( 0 \right)}/{\Phi}_2 \approx 1.02$ for low protein production rates and then decays linearly up to Φ_1_/Φ_1_ ≈ 0.98.

In the first set of experiments (magenta points in Fig. 3), and to a lesser degree in the second set of experiments (cyan points), the data points at the lowest production rate (i.e., at 0 μM IPTG) appear to deviate from the linear trend, and so the fits in Fig. 3a–c were calculated by excluding those data points (including them in the fit doesn’t affect the results, see Fig. S.21). The flow cytometry data suggest that the average fluorescent intensity of strain 1 from the induced RFP decreased over the course of the experiment at 0 μM IPTG, which may partly explain the deviation of the first magenta point in Fig. 3a from the linear trend via a reduction in protein production rate throughout the experiment at 0 μM IPTG. Another factor that may cause deviations from a linear trend is an increased gene-expression heterogeneity between cells in the absence of IPTG, a well-known property of the lac operon whose constituent parts we have used in our genetic constructs [47], which might confer heterogeneous growth rates to different cells in the population. Note that the normalized protein production rates of the two sets of experiments (magenta and cyan data points) are not directly comparable.

### Evaluation of the ratios Φ_1_/Φ_2_ and Φ_3_/Φ_4_

Consider the competition assay with strains 1 and 2 (the results are the same also for the competition assay between strains 3 and 4, after all subscripts are appropriately changed). For a given IPTG concentration *C*_*I*_, from Eq. (37) the growth rate of strain 1 is $g_1\left[ {k\left( {C_I} \right)} \right] = {\Phi}_1\left[ {k\left( {C_I} \right)} \right] \cdot \eta r\left( c \right)/\left( {1 + \gamma r\left( c \right)} \right)$ (where we have inserted explicitly the dependence on *r*(*c*), and *k*(*C*_*I*_) is the protein production rate induced by *C*_*I*_). On the other hand, the expression of the selective advantage for general values of *r*(*c*) is:

42 $$S = \frac{{\eta \cdot r\left( c \right)}}{{1 + \gamma \cdot r\left( c \right)}}\left( {{\Phi}_1 - {\Phi}_2} \right)$$

(in fact, if we only remove the assumption that *r*(*c*) ≈ 1, from Eq. (36a) we have $\dot m_\sigma /m_\sigma = \eta _\sigma r\left( c \right)\varphi _\sigma$ with *σ* = 1, 2 and the definition of *S* leads to this equation). Dividing *S* in Eq. (42) by *g*_1_, for any value of *r*(*c*) we obtain:

43 $$\frac{{S\left[ {k\left( {C_I} \right)} \right]}}{{g_1\left[ {k\left( {C_I} \right)} \right]}} = \frac{{{\Phi}_1\left[ {k\left( {C_I} \right)} \right] - {\Phi}_2\left[ {k\left( {C_I} \right)} \right]}}{{{\Phi}_1\left[ {k\left( {C_I} \right)} \right]}},$$

which is easily rearranged into:

44 $$\frac{{\Phi}_1}{{{\Phi}_2}}\left[ {k\left( {C_I} \right)} \right] = \frac{1}{{1 - S\left[ {k\left( {C_I} \right)} \right]/g_1\left[ {k\left( {C_I} \right)} \right]}}$$

(which are the values plotted in Fig. 3c). Notice again that this result does *not* depend on the assumption that *r*(*c*) = 1 at all times, i.e. Eq. (44) is valid for any value of *r*(*c*).

### Proteome fraction allocated to the inducible RFP

Because we did not measure RNA/protein ratios in our experiments, we can only estimate the values of *κ*^*t*^ and *κ*^*n*^ by taking them from the literature for *E. coli* strains grown at 30 °C in conditions similar to our experiments. Rosset et al. [48, 49] measured the RNA/protein ratio of several *E. coli* strains grown at 30 °C in M63 medium. Using their data and the relationship [22] *r* = *r*_0_ + *g*/*κ*^*t*^, where *r* is the RNA/protein ratio and *r*_0_ a constant, we can estimate the translational capacity as *κ*^*t*^ = 3.0 ± 0.5 μg protein/μg RNA · 1/h (mean ± SD). An estimate for the nutritional capacity *κ*^*n*^, instead, can be obtained via the equation [22] $g = g_{{\mathrm{max}}}\kappa ^n/\left( {\kappa ^n + \kappa ^t} \right)$, where *g*_max_ is the maximum growth rate obtainable by our strain at a given temperature (for us, 30 °C), when nutrients are abundant. Van Derlinden and Van Impe [50] report a maximum growth rate *g*_max_ ≈ 1.2 1/h for *E. coli* MG1655 grown at 30 °C in rich medium with glucose (no error estimate was reported). Solving for *κ*^*n*^ and using the growth rate value *g* measured for strain 1 in the absence of IPTG, we find *κ*^*n*^ = 1.2 ± 0.2 μg protein/μg RNA · 1/h. These values allow us to estimate $\gamma = \kappa ^n/\kappa ^t = 0.4 \pm 0.1$ and $\eta = \kappa ^n/\rho = 1.57 \pm 0.07$ 1/h using the value for *ρ* = 0.76 μg protein/μg RNA · 1/h reported in Scott et al. [22]. With these estimations, from the expression of the selective advantage in Eq. (42) we have that a 1% difference in proteome allocation for metabolism and growth between the two strains (i.e., Φ_1_ − Φ_2_ = 1%) leads to *S* ≈ 1.1 · 10^−2^. Finally, with these calculations we can estimate the maximum percentage of proteome max*φ*_*iRFP*_ and max*φ*_*iYFP*_ allocated at full expression to the production of, respectively, the inducible red and yellow proteins in our two experiments. In particular, for the first experiment we have ${\mathrm{max}}\varphi _{iRFP} = {\Phi}_1^{\left( 0 \right)} - {\Phi}_2 - \left( {1 + \gamma } \right)/\eta \cdot S_{105} = \left( {1 + \gamma } \right)/\eta \cdot \left( {S_0 - S_{105}} \right) \approx 1.1\%$ (where *S*_0_ and *S*_105_ are, respectively, the mean selection coefficients in the 0 μM and 105 μM IPTG treatments). For the experiment involving strains 3 and 4, using the same procedure we find max*φ*_*iYFP*_ ≈ 0.4%. Of course, given that we had to rely on measurements taken from the literature, these should be regarded as only rough estimates.

## Supplementary information

Supplementary Information

## Data Availability

The raw flow cytometry data studied in this work and the software used to analyze it are all available at the following GitHub repository: https://github.com/LeonardoPaccianiMori/CPR-model-experiment-data-analysis.
