# Supplementary material for: Constrained proteome allocation affects coexistence in models of competitive microbial communities
Source: ISME J. 2021 Jan 11;15(5):1458–77. doi: 10.1038/s41396-020-00863-0 (PMC8115080; doi:10.1038/s41396-020-00863-0)
Supplement: Supplementary file 1 — Supplementary Information [file 41396_2020_863_MOESM1_ESM.pdf]

Supplementary Information for "Constrained proteome allocation affects coexistence in models of competitive microbial communities"

Leonardo Pacciani-Mori,[1,2] Samir Suweis,[1] Amos Maritan,[1] and Andrea Giometto[3]

[1] *Department of Physics and Astronomy "Galileo Galilei", University of Padua Via Marzolo 8 35131, Padua (Italy)*

[2] *Department of Physics, Harvard University 17 Oxford St, Cambridge 02138 MA*

[3] *School of Civil and Environmental Engineering, Cornell University 220 Hollister Dr, Ithaca 14853 NY*

CONTENTS

I. The consumer-proteome-resource model 2

A. Biological interpretation of ξ_i in Eq (4b) 2

B. Definition of yield in the consumer-proteome-resource model 2

C. Dynamics of $\varphi_{\sigma i}$ – complete expression of Eq (19) 2

D. Conditions for coexistence – derivation of Eq (28) 3

E. Further differences between the consumer-proteome-resource model and Posfai *et al.* 3

F. Parameters used in the simulations 4

II. Experiment 5

A. Computation of the normalized protein production rate 5

B. Data 5

1. Time series 5

2. OD curves and growth rates 6

3. Plasmid-induced fitness advantage 6

References 6

Figures and Tables 7

I. THE CONSUMER-PROTEOME-RESOURCE MODEL

A. Biological interpretation of ξ_i in Eq (4b)

Scott et al. [1, Supporting Online Material] give a microscopic interpretation of the nutritional capacity κ_i^n by stating that the growth rate of a microbial species is given by $g = qJ$, where J is the uptake rate of the (only) resource per unit biomass, and q is a proportionality constant that depends on the properties of the nutrient (e.g., how much energy its metabolization can generate). They then assume that there is only one bottleneck enzyme E for the growth of the microbial species, and write:

$$J = k_E r(c)\varphi_E \ , \tag{S.1}$$

where k_E is the maximal catalytic rate of enzyme E, $r(c)$ is Monod's function, and φ_E is the fraction of the proteome occupied by the enzyme E. Compared to our formalism in the general case of N_S species and N_R resources (so that $J \to J_{\sigma i}$ and $g \to g_\sigma$), we can identify $r(c) \to r_i(c_i)$, $\varphi_E \to \varphi_{\sigma i}$, $q \to \chi_{\sigma i}$ and finally $k_E \to \xi_i$.

B. Definition of yield in the consumer-proteome-resource model

Let us consider the consumer-proteome-resource model with a single species and a single resource which is only supplied initially (i.e., $s = 0$):

$$\dot{m} = m(\eta r(c)\varphi - q) \ , \tag{S.2a}$$

$$\dot{c} = -\xi r(c) m\varphi \ , \tag{S.2b}$$

$$\varphi(1 + \gamma r(c) = \Phi \ . \tag{S.2c}$$

If we neglect the maintenance cost, i.e. $q \approx 0$, we can rewrite Eq (S.2a) as:

$$\frac{\dot{m}}{m} = \eta r(c)\varphi \qquad \Rightarrow \qquad \frac{dm}{m} = \eta r(c)\varphi dt \ , \tag{S.3}$$

and Eq (S.2b) as

$$\frac{dc}{m\varphi} = -\xi r(c) dt \ . \tag{S.4}$$

Plugging Eq (S.4) into Eq (S.3) we get:

$$\frac{dm}{m} = -\eta\varphi \cdot \frac{dc}{m\varphi\xi} \ , \tag{S.5}$$

and therefore the yield, defined as the cell biomass per gram of resource, is given by:

$$Y := \left|\frac{dm}{dc}\right| = \frac{\eta}{\xi} \ . \tag{S.6}$$

C. Dynamics of $\varphi_{\sigma i}$ – complete expression of Eq (19)

In components, Eq (19) is written as follows:

$$\dot{\varphi}_{\sigma i} = \frac{1}{\tau_\sigma}\frac{\partial g_\sigma}{\partial \varphi_{\sigma i}} - \frac{\partial F_\sigma / \partial \varphi_{\sigma i}}{\sum_{k=1}^{N_R} (\partial F_\sigma / \partial \varphi_{\sigma k})^2} \sum_{j=1}^{N_R} \left(\frac{1}{\tau_\sigma}\frac{\partial g_\sigma}{\partial \varphi_{\sigma j}}\frac{\partial F_\sigma}{\partial \varphi_{\sigma j}} + \dot{c}_j \frac{\partial F_\sigma}{\partial c_j} \right) \ . \tag{S.7}$$

To make sure that $\varphi_{\sigma i}$ doesn't become negative, we use the following approach. We rewrite Eq (S.7) in terms of an auxiliary variable $\psi_{\sigma i}$ instead of $\varphi_{\sigma i}$:

$$\dot{\psi}_{\sigma i} = \frac{1}{\tau_\sigma}\frac{\partial g_\sigma}{\partial \psi_{\sigma i}} - \frac{\partial F_\sigma / \partial \psi_{\sigma i}}{\sum_{k=1}^{N_R} (\partial F_\sigma / \partial \psi_{\sigma k})^2} \sum_{j=1}^{N_R} \left(\frac{1}{\tau_\sigma}\frac{\partial g_\sigma}{\partial \psi_{\sigma j}}\frac{\partial F_\sigma}{\partial \psi_{\sigma j}} + \dot{c}_j \frac{\partial F_\sigma}{\partial c_j} \right) \ , \tag{S.8}$$

and then we define $\varphi_{\sigma i} = \mathcal{F}(\psi_{\sigma i})$, where $\mathcal{F}$ is a positive function, i.e. $\mathcal{F}(x) > 0\ \forall x$. This way, Eq (S.8) becomes:

$$\dot{\varphi}_{\sigma i} = \mathcal{F}'(\psi_{\sigma i})^2 \left[\frac{1}{\tau_\sigma} \frac{\partial g_\sigma}{\partial \varphi_{\sigma i}} - \frac{\partial F_\sigma / \partial \varphi_{\sigma i}}{\sum_{k=1}^{N_R} \left(\partial F_\sigma / \partial \varphi_{\sigma k}\right)^2 \left(\mathcal{F}'(\psi_{\sigma k})\right)^2} \sum_{j=1}^{N_R} \left(\frac{\mathcal{F}'(\psi_{\sigma j})^2}{\tau_\sigma} \frac{\partial g_\sigma}{\partial \varphi_{\sigma j}} \frac{\partial F_\sigma}{\partial \varphi_{\sigma j}} + \dot{c}_j \frac{\partial F_\sigma}{\partial c_j} \right) \right] . \tag{S.9}$$

If we choose $\mathcal{F}(x) = x^2/4$ (any other choice leads to qualitatively identical results), so that $\mathcal{F}'(\psi_{\sigma i})^2 = \varphi_{\sigma i}$, and taking into account that:

$$\frac{\partial g_\sigma}{\partial \varphi_{\sigma i}} = \eta_{\sigma i} r_i(c_i) , \qquad \frac{\partial F_\sigma}{\partial \varphi_{\sigma i}} = 1 + \gamma_{\sigma i} r_i(c_i) , \qquad \frac{\partial F_\sigma}{\partial c_i} = \varphi_{\sigma i} \gamma_{\sigma i} \frac{K_i}{(c_i + K_i)^2} , \tag{S.10}$$

we have that the final expressions of the equation for $\dot{\varphi}_{\sigma i}$ (i.e., Eq (19)), in components, is:

$$\dot{\varphi}_{\sigma i} = \varphi_{\sigma i} \left[\frac{\eta_{\sigma i} r_i(c_i)}{\tau_\sigma} - \frac{(1 + \gamma_{\sigma i} r_i(c_i))}{\sum_{k=1}^{N_R} \varphi_{\sigma k} (1 + \gamma_{\sigma k} r_k(c_k))^2} \sum_{j=1}^{N_R} \varphi_{\sigma j} \left(\frac{\eta_{\sigma j} r_j(c_j)}{\tau_\sigma} (1 + \gamma_{\sigma j} r_j(c_j)) + \gamma_{\sigma j} \frac{K_j}{(c_j + K_j)^2} \dot{c}_j \right) \right] . \tag{S.11}$$

D. Conditions for coexistence – derivation of Eq (28)

Plugging Eq (21) into Eq (20b) we obtain:

$$s_i \frac{\kappa_i^n}{\xi_i} = \frac{1}{\Theta} \sum_{\sigma=1}^{N_S} m_\sigma^* \varphi_{\sigma i}^* , \tag{S.12}$$

and by summing this equation over i on both sides, we get

$$\sum_{i=1}^{N_R} s_i \frac{\kappa_i^n}{\xi_i} = \sum_{\sigma=1}^{N_S} m_\sigma^* \frac{1}{\Theta} \sum_{i=1}^{N_R} \varphi_{\sigma i}^* = \sum_{\sigma=1}^{N_S} m_\sigma^* \rho_\sigma q_\sigma . \tag{S.13}$$

If we now define

$$\hat{s}_i := \frac{s_i \kappa_i^n / \xi_i}{\sum_{j=1}^{N_R} s_j \kappa_j^n / \xi_j} , \qquad \hat{\varphi}_{\sigma i}^* := \frac{\varphi_{\sigma i}^*}{\sum_{j=1}^{N_R} \varphi_{\sigma j}^*} \tag{S.14}$$

(so that $\sum_i \hat{s}_i = \sum_i \hat{\varphi}_{\sigma i} = 1$), Eq (S.12) becomes:

$$\hat{s}_i = \sum_{\sigma=1}^{N_S} \frac{m_\sigma^* \rho_\sigma q_\sigma}{\sum_{\lambda=1}^{N_S} m_\lambda^* \rho_\lambda q_\lambda} \hat{\varphi}_{\sigma i}^* , \tag{S.15}$$

that is exactly Eq (28) of the Main Text once we define

$$z_\sigma := \frac{m_\sigma^* \rho_\sigma q_\sigma}{\sum_{\lambda=1}^{N_S} m_\lambda^* \rho_\lambda q_\lambda} , \tag{S.16}$$

which are indeed positive coefficients such that $\sum_{\sigma=1}^{N_S} z_\sigma = 1$.

E. Further differences between the consumer-proteome-resource model and Posfai *et al.*

The main difference between our consumer-proteome-resource model and classic consumer-resource theory is the introduction of the proteome finiteness constraint, i.e. Eq (4c). The functional form of this constraint is rooted in previous experimental results on the link between proteome allocation and microbial growth [1], and allows us to link the macroscopic parameters describing community dynamics (e.g., growth rates) to microscopic parameters related to gene expression and proteome allocation. We found that the existence of such a constraint implies that the proteome fractions $\varphi_{\sigma i}$ must be dynamic variables rather than fixed parameters, which is another difference with respect to

classic consumer-resource theory, and is in agreement with the well known fact that microbial species can vary their uptake of different resources based on the concentration of such resources in the environment.

Note that the constraint introduced here is substantially different from previous constraints considered in consumer-resource models with metabolic trade-offs, like e.g. the one introduced in Posfai *et al.* Therein, the metabolic strategies (whose analogues in our framework are the proteome fractions $\varphi_{\sigma i}$) were assumed to be fixed, and subject to a constraint that in our notation would read $\sum_{i=1}^{N_R} \varphi_{\sigma i} = \Phi$, which assumes $\Phi_\sigma = \Phi \; \forall \sigma$ and *does not* involve the resources' concentrations through $r_i(c_i)$. The constraint in Eq (4c) reduces to this one in very particular cases, e.g. $r_i(c_i) \approx 0$ or $\gamma_{\sigma i} \approx 0$. The constraint introduced in Posfai *et al.* is phenomenological and was meant to account for the fact that species have a limited amount of energy that they can devote to nutrient uptake and metabolization. However, it does not account for experimentally-verified tradeoffs between protein synthesis and nutrient influx in microbes that lead to the constraint in Eq (4c), and change the overall dynamics. Our work thus builds on the literature on consumer-resource models and identifies an experimentally-supported way to introduce metabolic tradeoffs and constraints that are appropriate for modeling microbial communities.

One of the conditions for coexistence in the consumer-proteome-resource model can be represented using the same graphical representation introduced by Posfai *et al.* (section I D). However, given that proteome fractions are dynamic variables in the consumer-proteome-resource model, a major difference with Posfai *et al.* is that the condition derived here involves the proteome fractions at *stationarity*. As a consequence, in the consumer-proteome-resource model a community with more species than resources can coexist, unlike in traditional consumer-resource models, and coexistence can occur even if the rescaled resource supply rate is initially outside of the convex hull of the rescaled proteome fractions.

Finally, introducing dynamic proteome fractions $\varphi_{\sigma i}$ in consumer-resource models through the adaptive process described in I C allows the consumer-proteome-resource model to describe phenomena that classic consumer-resource theory cannot. The most prominent example is that of diauxic shifts: Figure S.2 shows a simulation of our model for a system with one species, $N_S = 1$, and two resources, $N_R = 2$, with parameters set to reproduce the growth of *E. coli* on glucose and a second resource with lower nutritional quality and maximum catalytic rate of the enzymes used to metabolize it. The temporal increase in the species' biomass according to the consumer-proteome-resource model in this context resembles a diauxic shift in which glucose is consumed first. Additionally, Figure S.2b-c shows that when the concentration of the most favorable resource is large, the proteome fraction dedicated to the uptake of that resource grows at the expense of the proteome fraction dedicated to the other resource. Consumption of the less favorable resource occurs only after the most favorable one is at very small concentrations. A classic consumer-resource model, instead, would predict a single exponential growth curve in these settings.

F. Parameters used in the simulations

Figure 5: The parameters used to run the simulations shown in Figure 5 of the Main Text were drawn randomly from the following distributions: $m_\sigma(0) \in \mathcal{U}[1,5]$ μg of biomass/mL (which is in the order of $\approx 10^6$ cells/mL, assuming an average mass of $\approx 10^{-12}$ g for an *E. coli* cell), with $\mathcal{U}$ the uniform distribution, $c_i(0) \in \mathcal{U}[0.5 \cdot 10^4, 1.5 \cdot 10^4]$ μg of resource/mL (which is in the order of a concentration of roughly 1% w/v), $\kappa_i^n \in \mathcal{U}[10,50]$ μg protein/μg RNA $\cdot$ 1/h, $\kappa_\sigma^t \in \mathcal{U}[1,5]$ μg protein/μg RNA $\cdot$ 1/h (so that $\gamma_{\sigma i} \sim O(0.1)$), $\rho_\sigma \in \mathcal{U}[0.5, 0.9]$ μg protein/μg RNA, $s_i \in \mathcal{U}[0.5 \cdot 10^4, 2 \cdot 10^4]$ μg of resource/mL $\cdot$ 1/h, $\xi_i \in \mathcal{U}[10^3, 1.5 \cdot 10^3]$ 1/h (which corresponds to ≈ 0.35 1/s, the catalytic rate of the slowest enzyme involved in the glycolytic pathway of *E. coli*, as reported by Gameiro et al. [2]), $K_i \in \mathcal{U}[5 \cdot 10^2, 10^3]$ μg/mL (which is in the order of $\approx 8 \cdot 10^2$ μg/L as reported by Seen et al. [3] for *E. coli* at 30°C), $\tau_\sigma \in \mathcal{U}[10^4, 5 \cdot 10^4]$ 1/h (so that $\tau_\sigma \gg 1$), $\Phi_\sigma \in \mathcal{U}[0.45, 0.55]$, $\varphi_{\sigma i}$ were drawn randomly so that they satisfy $\sum_i \varphi_{\sigma i}(1 + \gamma_{\sigma i} r_i(c_i(0))) = \Phi_\sigma$, and $\Theta = 5$.

Figure 6: The parameters used to run the simulations shown in Figure 6 of the Main Text were drawn randomly from the following distributions: $m_\sigma(0) \in \mathcal{U}[1,5]$ μg of biomass/mL, $c_i(0) \in \mathcal{U}[0.5 \cdot 10^4, 1.5 \cdot 10^4]$ μg of resource/mL, $\kappa_i^n \in \mathcal{U}[5,10]$ μg protein/μg RNA $\cdot$ 1/h, $\kappa_\sigma^t \in \mathcal{U}[1,5]$ μg protein/μg RNA $\cdot$ 1/h (so that $\gamma_{\sigma i} \sim O(2)$), $\rho_\sigma \in \mathcal{U}[0.6, 0.8]$ μg protein/μg RNA, $s_i \in \mathcal{U}[0.5 \cdot 10^4, 10^4]$ μg of resource/mL $\cdot$ 1/h, $\xi_i \in \mathcal{U}[10^3, 1.5 \cdot 10^3]$ 1/h, $K_i \in \mathcal{U}[5 \cdot 10^2, 10^3]$ μg/mL, $\tau_\sigma \in \mathcal{U}[10^4, 5 \cdot 10^4]$ 1/h (so that $\tau_\sigma \gg 1$), $\Phi_\sigma \in \mathcal{U}[0.45, 0.55]$, $\varphi_{\sigma i}$ were drawn randomly so that they satisfy $\sum_i \varphi_{\sigma i}(1 + \gamma_{\sigma i} r_i(c_i(0))) = \Phi_\sigma$, and $\Theta = 5$.

Figure 7: The parameters used to run the simulations shown in Figure 7 of the Main Text were drawn randomly from the following distributions: $m_\sigma(0) \in \mathcal{U}[1,5]$ μg of biomass/mL, $c_i(0) \in \mathcal{U}[0.5 \cdot 10^4, 1.5 \cdot 10^4]$ μg of resource/mL, $\kappa_i^n \in \mathcal{U}[2,5]$ μg protein/μg RNA $\cdot$ 1/h, $\kappa_\sigma^t \in \mathcal{U}[1,4]$ μg protein/μg RNA $\cdot$ 1/h (so that $\gamma_{\sigma i} \sim O(1)$), $\rho_\sigma \in \mathcal{U}[0.6, 0.8]$ μg protein/μg RNA, $s_i \in \mathcal{U}[0.5 \cdot 10^4, 2 \cdot 10^4]$ μg of resource/mL $\cdot$ 1/h, $\xi_i \in \mathcal{U}[10^3, 1.5 \cdot 10^3]$ 1/h,

$K_i \in \mathcal{U}[5 \cdot 10^2, 10^3]$ μg/mL, $\tau_\sigma \in \mathcal{U}[1,5]$ 1/h, $\Phi_\sigma \in \mathcal{U}[0.45, 0.55]$, $\varphi_{\sigma i}$ were drawn randomly so that they satisfy $\sum_i \varphi_{\sigma i}(1 + \gamma_{\sigma i} r_i(c_i(0))) = \Phi_\sigma$, and $\Theta = 5$.

II. EXPERIMENT

A. Computation of the normalized protein production rate

Let us call $k(C_I)$ the production rate of the induced fluorescent protein when cells are exposed to an IPTG concentration of C_I, and let us call d_{FP} the protein degradation rate. If we call $\mathcal{I}$ the fluorescent intensity of a cell due to the induced protein, between two consecutive cell divisions $\mathcal{I}$ will satisfy:

$$\frac{d\mathcal{I}}{dt} = k(C_I) - d_{FP}\mathcal{I} \ , \tag{S.17}$$

whose solution is:

$$\mathcal{I}(t) = \mathcal{I}_0 e^{-d_{FP}t} + \frac{k(C_I)}{d_{FP}}\left(1 - e^{-d_{FP}t}\right) \ . \tag{S.18}$$

If we now call τ the doubling time and we take into account that the $\mathcal{I}$ is halved after every cellular division, the fluorescent intensity $\mathcal{I}_i$ at the i-th division will be:

$$\mathcal{I}_1 = \mathcal{I}_0 e^{-d_{FP}\tau} + \frac{k(C_I)}{d_{FP}}(1 - e^{-d_{FP}\tau}) \tag{S.19a}$$

$$\mathcal{I}_2 = \frac{\mathcal{I}_1}{2} e^{-d_{FP}\tau} + \frac{k(C_I)}{d_{FP}}(1 - e^{-d_{FP}\tau}) = \frac{\mathcal{I}_0}{2} e^{-d_{FP}2\tau} + \frac{k(C_I)}{d_{FP}}(1 - e^{-d_{FP}\tau})\left(1 + \frac{1}{2}e^{-d_{FP}\tau}\right) \tag{S.19b}$$

$$\mathcal{I}_3 = \frac{\mathcal{I}_2}{2} e^{-d_{FP}\tau} + \frac{k(C_I)}{d_{FP}}(1 - e^{-d_{FP}\tau}) = \frac{\mathcal{I}_0}{4} e^{-d_{FP}3\tau} + \frac{k(C_I)}{d_{FP}}(1 - e^{-d_{FP}\tau})\left(1 + \frac{1}{2}e^{-d_{FP}\tau} + \frac{1}{4}e^{-2d_{FP}\tau}\right) \tag{S.19c}$$

$$\vdots$$

$$\mathcal{I}_i = \frac{\mathcal{I}_0}{2^{i-1}} e^{-d_{FP}\tau i} + \frac{k(C_I)}{d_{FP}}(1 - e^{-d_{FP}\tau}) \sum_{\ell=1}^{i} \frac{e^{-(\ell-1)d_{FP}\tau}}{2^{\ell-1}} \ . \tag{S.19d}$$

If we now define $\tau i = t$, and take into account that $\tau = \ln 2/g$ (with g the cell's growth rate), and we use the fact that

$$\sum_{\ell=1}^{i} \frac{e^{-(\ell-1)d_{FP}\tau}}{2^{\ell-1}} = \frac{2e^{d_{FP}\tau}(1 - 2^{-i}e^{-d\tau i})}{2e^{d_{FP}\tau} - 1} \ , \tag{S.20}$$

and we can rewrite Eq (S.19d) as:

$$\mathcal{I}(t) = 2\mathcal{I}_0 e^{-gt\left(1+\frac{d_{FP}}{g}\right)} + \frac{k(C_I)}{d_{FP}}\left[1 - e^{-gt\left(1+\frac{d_{FP}}{g}\right)}\right]\left(1 - \frac{1}{2^{1+\frac{d_{FP}}{g}} - 1}\right) \tag{S.21}$$

which is Eq (30) in the Main Text.

B. Data

1. *Time series*

Figures S.19 and S.20 show the time series of $\ln f/(1-f)$ for both the experiments shown in Figure 3 of the Main Text. We can see that the trend of the first day is in contrast with the rest of the experiment (i.e., $\ln f/(1-f)$ decreases in the first 24h and then increases, or vice versa). We found out that this is due to the fact at 0h some flow-cytometry data are very close to the experimental noise, and therefore the two strains are difficult to resolve. The selective advantages have therefore been computed by fitting each curve with a linear function between day 1 and day 6 (i.e., between 24h and 144h).

2. OD curves and growth rates

Figures S.15 and S.17 show the growth curves of strains 1 and 3 at different IPTG concentrations. Figures S.16 and S.18, show the values of the growth rates obtained from these curves. Note that the difference in growth rate of the IPTG-inducible strains between the maximum and minimum concentrations of IPTG is small (about 0.01 1/h for strain 1 based on the selection coefficient measurements), and thus is barely noticeable in the growth rates estimated from the OD growth curves, due to the poor sensitivity of this assay compared to the competition assays presented in the main text.

3. Plasmid-induced fitness advantage

Strain 1 has a fitness advantage over strain 2 in the absence of IPTG (and the same is true for strains 3 and 4). We hypothesize that this advantage may be due to the additional copy of *lacI* carried by strain 1 on the plasmid. This hypothesis is corroborated by additional competition assays that we performed between different combinations of the ancestor strains and the strains carrying the plasmids using the same experimental protocol shown in Materials and Methods. These competition assays show that:

1. The ancestor strains 0Y and 0R, which constitutively express respectively mVenus and mKate2Hyb but do not posses any plasmids, have the same fitness at all concentrations of IPTG (see Figures S.3 and S.4).
2. Strain 1 has a fitness advantage over strain 0R, the ancestor of strain 2, in the absence of IPTG. Increasing the concentration of IPTG, the fitness advantage of strain 1 over strain 0R is reduced, until strain 0R grows faster than strain 1 at 105 μM IPTG (see Figures S.5 and S.6).
3. Strain 3 has a fitness advantage over strain strain 0Y, the ancestor of strain 4, in the absence of IPTG. Increasing the concentration of IPTG, the fitness advantage of strain 3 over strain 0Y is reduced but is never enough to allow 0Y to outcompete strain 3 (see Figures S.7 and S.8).
4. Strains 2 and 4 have a slight fitness advantage over their ancestors 0R and 0Y. Such an advantage is much smaller than the one that strains 1 and 3 have over their ancestor in the absence of IPTG (see Figures S.9, S.10, S.11 and S.12). A competition assay between strain 2 and 4 shows that they have the same fitness (see Figures S.13 and S.14).

Taken together, these data support the hypothesis that the extra copy of *lacI* in the plasmids is beneficial when strains that carry such plasmids are grown in the absence of IPTG. Furthermore, the fact that strain 3 is not outcompeted by strain 0Y at high concentrations of IPTG, and that instead 1 is outcompeted by 0R, suggests that the fitness cost of producing mVenus is smaller than the fitness cost of producing mCherry, which is in line with the observation that strain 3 is fitter than strain 4 at all concentrations of IPTG, as seen in Figure 3a of the Main Text.

[1] M. Scott, C. W. Gunderson, E. M. Mateescu, Z. Zhang, and T. Hwa, Interdependence of cell growth and gene expression, Science **330**, 10.1177/42.6.8189037 (2010).

[2] D. Gameiro, M. Pérez-Pérez, G. Pérez-Rodríguez, G. Monteiro, N. F. Azevedo, and A. Lourenço, Computational resources and strategies to construct single-molecule metabolic models of microbial cells, Briefings in Bioinformatics **17**, 863 (2015).

[3] H. Senn, U. Lendenmann, M. Snozzi, G. Hamer, and T. Egli, The growth of escherichia coli in glucose-limited chemostat cultures: a re-examination of the kinetics, Biochimica et Biophysica Acta (BBA) - General Subjects **1201**, 424 (1994).

Strain or plasmid name	Genotype/description	Name in collection
Plasmid pR	pTrc99A with mCherry introduced in the multiple cloning site. Amp^R, $lacI^Q$, pBR322 origin, pTrc_mCherry	pbAG3
Plasmid pY	pTrc99A with Venus YFP introduced in the multiple cloning site. Amp^R, $lacI^Q$, pBR322 origin, pTrc_Venus YFP	pbAG2
Plasmid pAMP	Largest digestion of pR with restriction enzyme SphI, ligated with ligase. Amp^R, pBR322 origin	pbAG9
Strain 0R	Markerless, chromosomally integrated insertion attTN7::pRpsL_mKate2Hyb	bAG11
Strain 0Y	Markerless, chromosomally integrated insertion attTN7::pRNA1_mVenus	bAG13
Strain 1	0Y transformed with pR	bAG17
Strain 2	0R transformed with pAMP	bAG25
Strain 3	0R transformed with pY	bAG16
Strain 4	0Y transformed with pAMP	bAG24

TABLE S.1: Information on the strains and plasmids used in our experiments

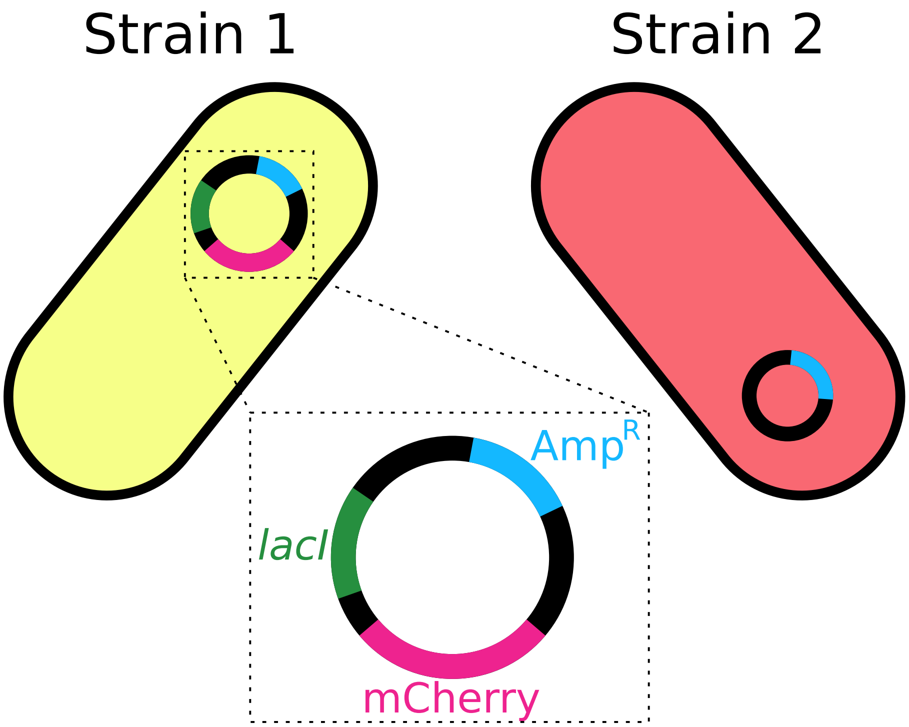

(a) Strains used in the first experiment (magenta points in Figure 3 of the Main Text).

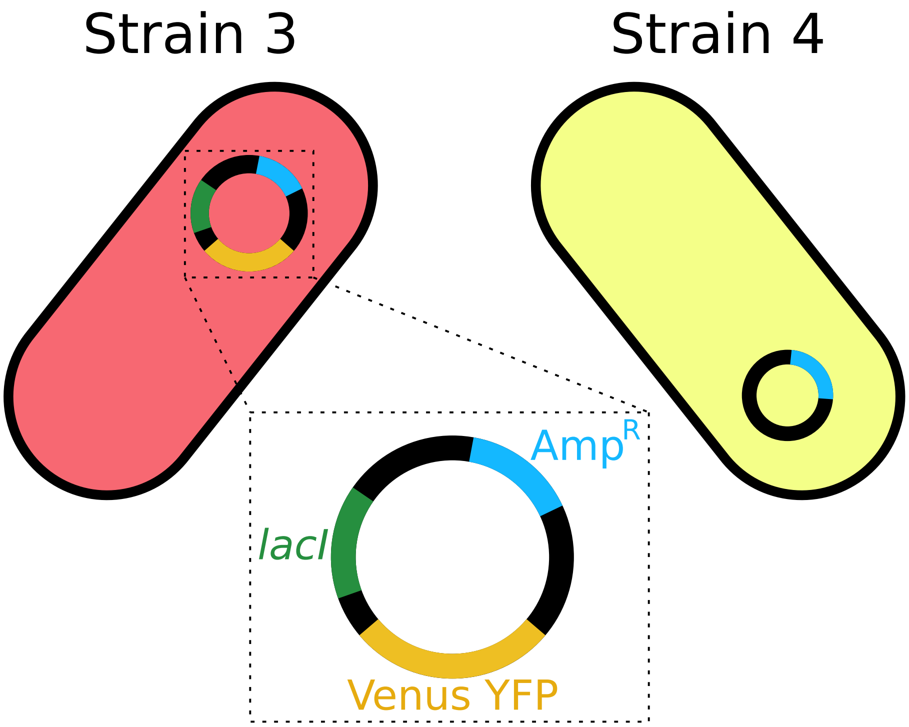

(b) Strains used in the second experiment (cyan points in Figure 3 of the Main Text).

FIG. S.1: Schematic representation of the strains used in both our experiments.

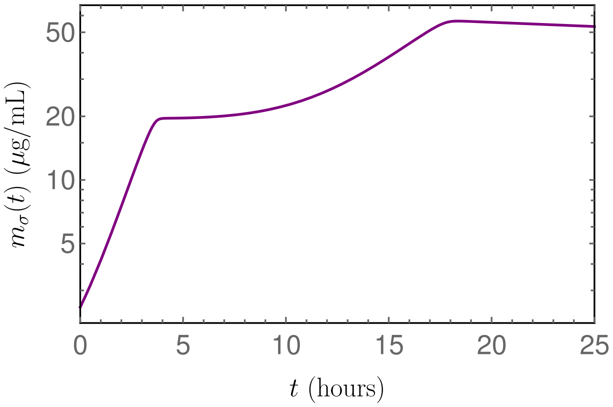

(a) Time evolution the species' biomass $m(t)$

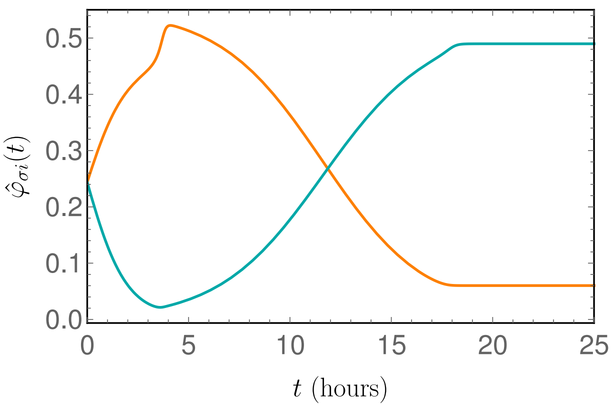

(b) Time evolution the species' rescaled proteome fractions $\hat{\varphi}_i$

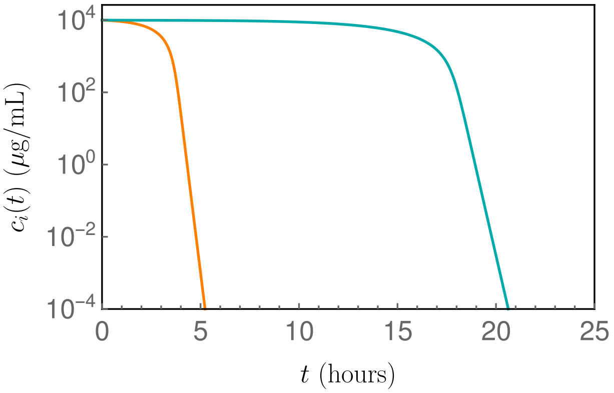

(c) Time evolution the resources' concentrations $c_i(t)$

FIG. S.2: Diauxic shifts as described by the consumer-proteome-resource model. The model parameters are set to reproduce the growth of *E. coli* on glucose (resource $i = 1$) and a second resource with lower nutritional capacity and maximum catalytic rate of the enzymes used to metabolize it. The parameters used are: $m(0) = 2.5$ μg of biomass/mL, $\vec{c}(0) = (10^4, 10^4)$ μg of resource/mL, $\vec{\kappa}^n = (1, 0.25)$ μg protein/μg RNA $\cdot$1/h, $\kappa^t = 4.5$ μg protein/μg RNA $\cdot$1/h, $\rho = 0.6$ μg protein/μg RNA, $s_i = 0$ μg of resource/mL $\cdot$1/h (i.e., both resources are supplied only initially), $\vec{\xi} = (1000, 100)$ 1/h, $\vec{K} = (1000, 500)$ μg/mL, $\tau = 1$ 1/h, $\Phi = 0.55$, $\Theta = 100$ and φ_i were chosen so that $\hat{\varphi}_1 = \hat{\varphi}_2$ and $\sum_{i=1,2} \varphi_i(1 + \gamma_i r_i(c_i(0)) = \Phi$.

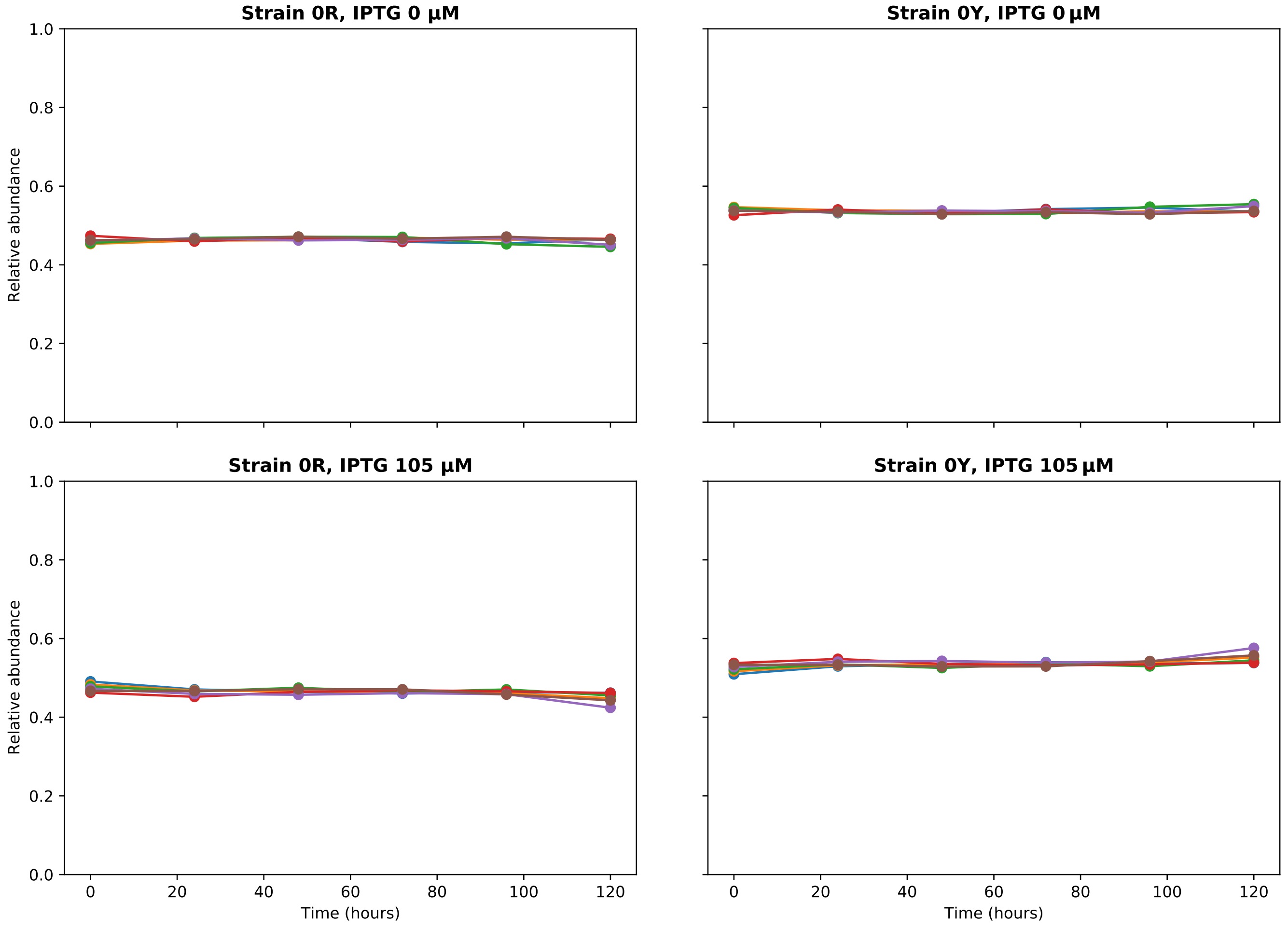

FIG. S.3: Competition assays between strains 0R and 0Y. These assays were done with the same experimental protocol shown in the Methods section (without adding ampicillin to the culture medium since both strains do not carry any plasmid and therefore are not ampicillin resistant).

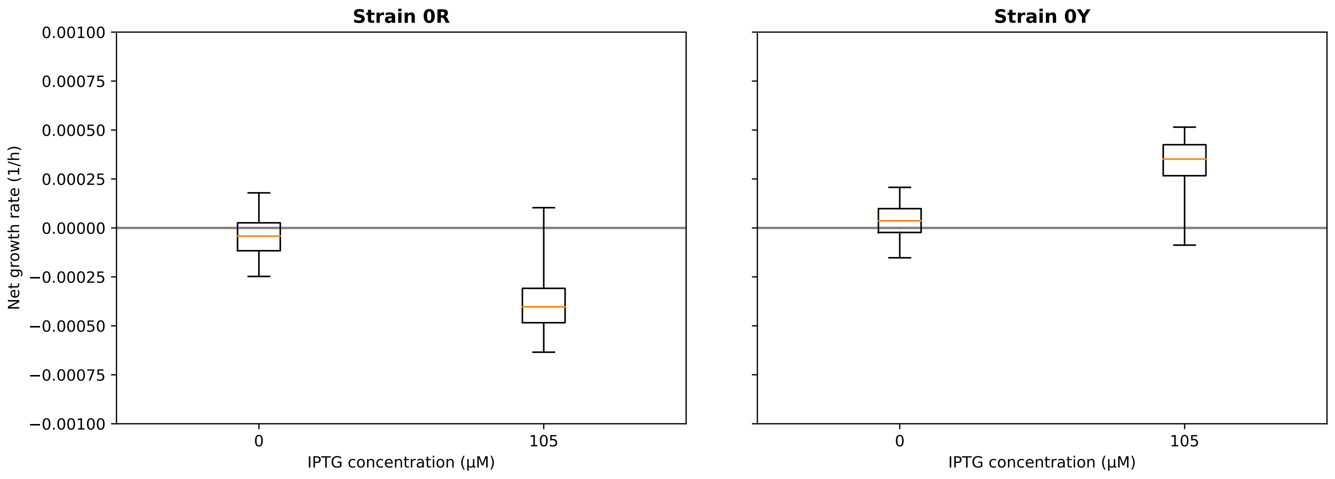

FIG. S.4: Boxplot of the growth rates of strains 0R and 0Y, computed by fitting the time series shown in Figure S.3.

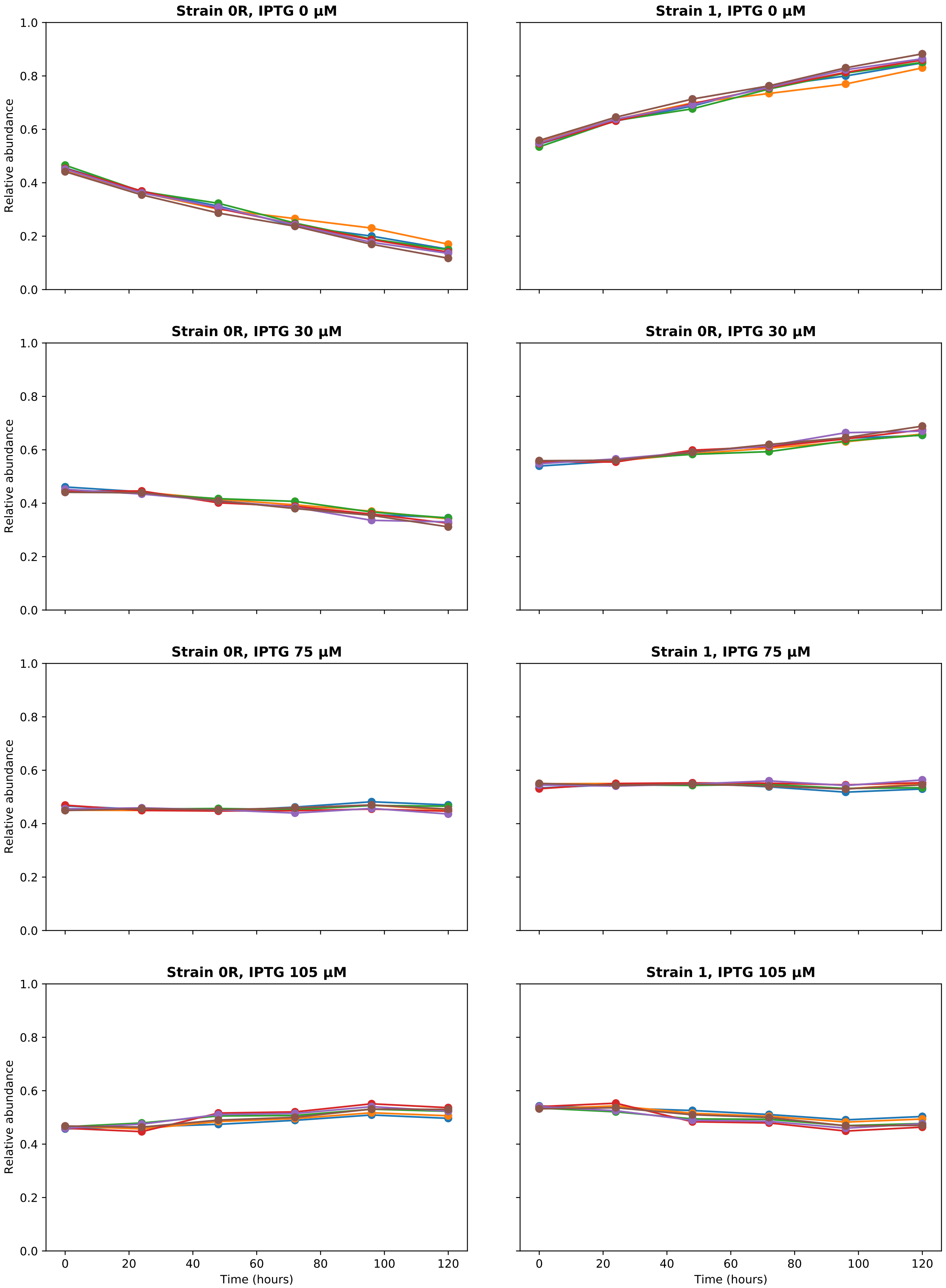

FIG. S.5: Competition assays between strains 0R and 1. These assays were done with the same experimental protocol shown in the Methods section (without adding ampicillin to the culture medium since strain 0R is not ampicillin resistant).

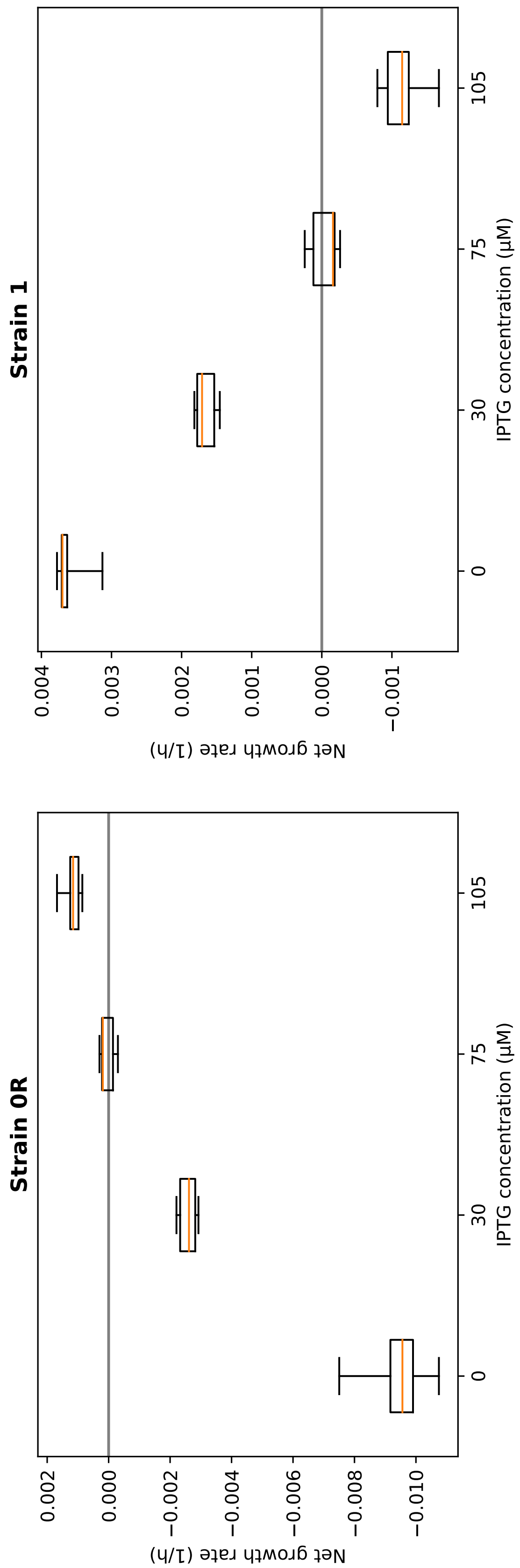

FIG. S.6: Boxplot of the growth rates of strains 0R and 1, computed by fitting the time series shown in Figure S.5. As we can see, in absence of IPTG strain 1 has a fitness advantage over 0R, which is reduced as the concentration of IPTG increases.

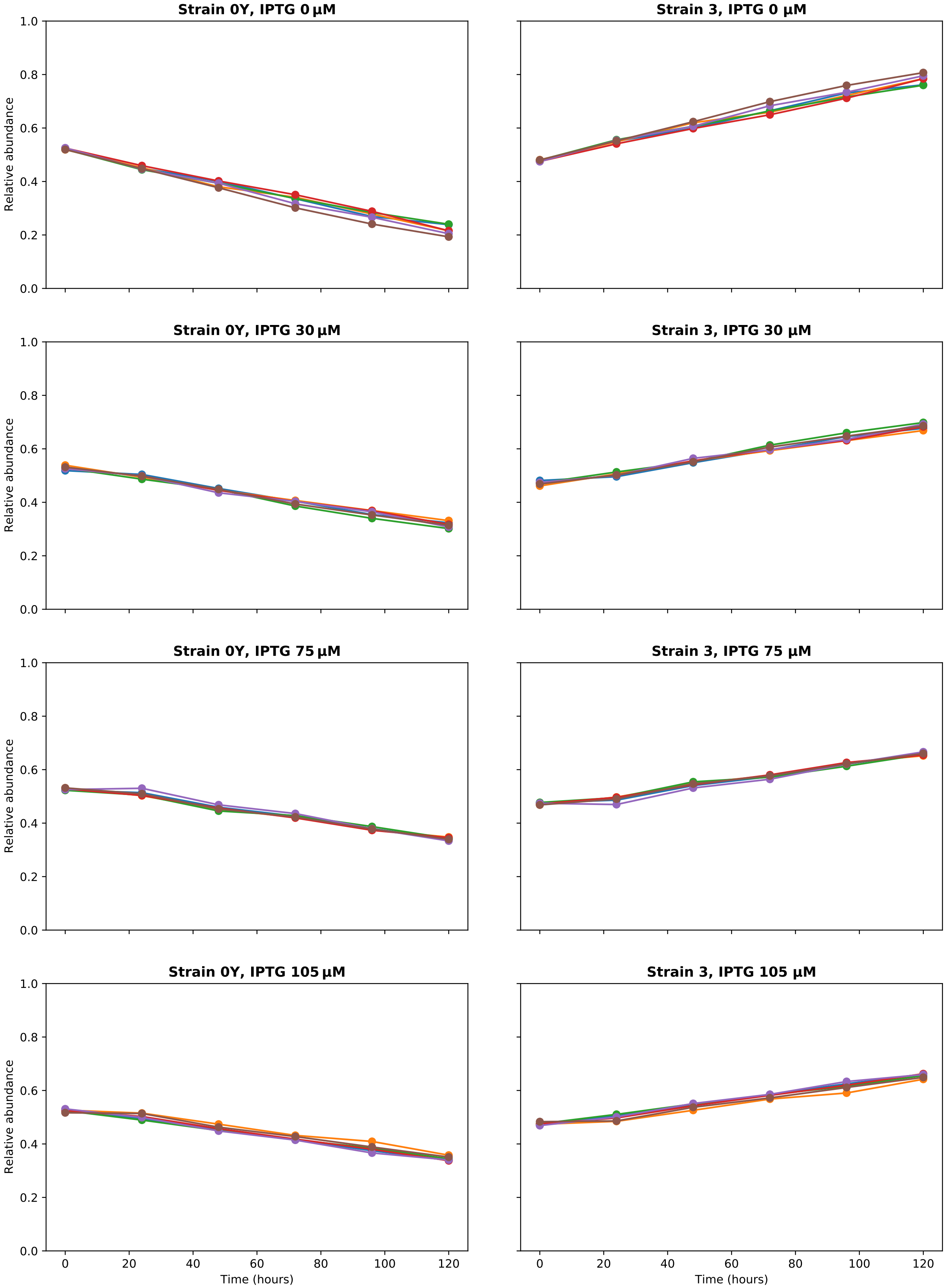

FIG. S.7: Competition assays between strains 0Y and 3. These assays were done with the same experimental protocol shown in the Methods section (without adding ampicillin to the culture medium since strain 0Y is not ampicillin resistant).

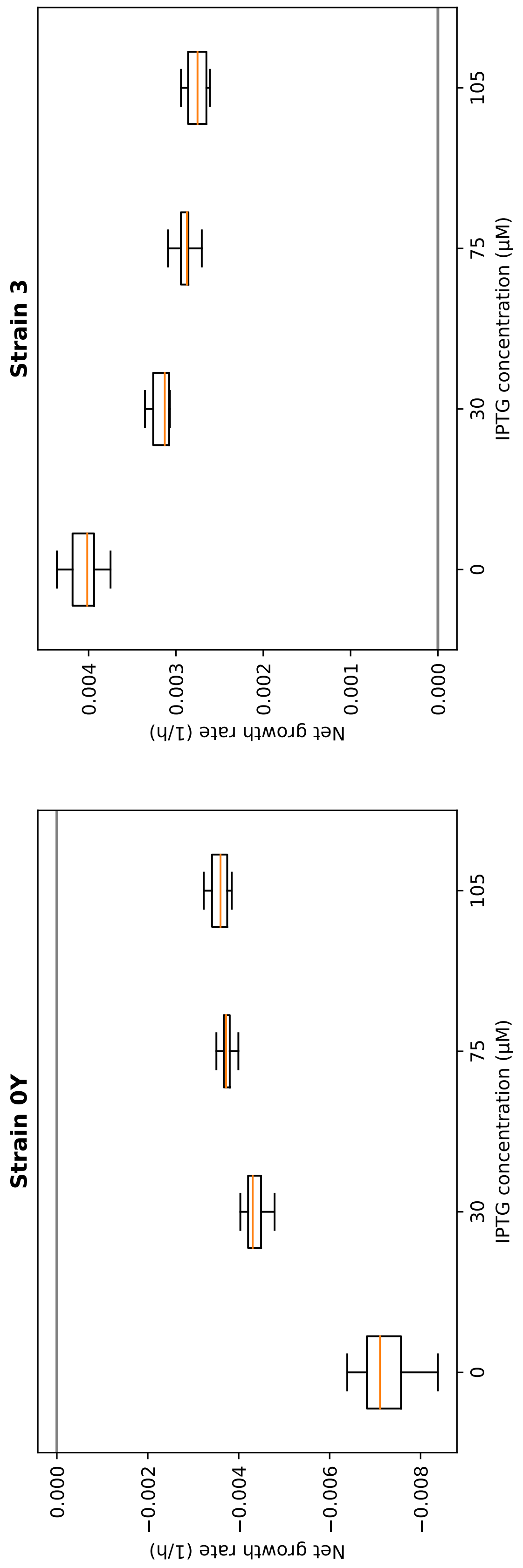

FIG. S.8: Boxplot of the growth rates of strains 0Y and 3, computed by fitting the time series shown in Figure S.7. As we can see, strain 3 always has a fitness advantage over 0Y.

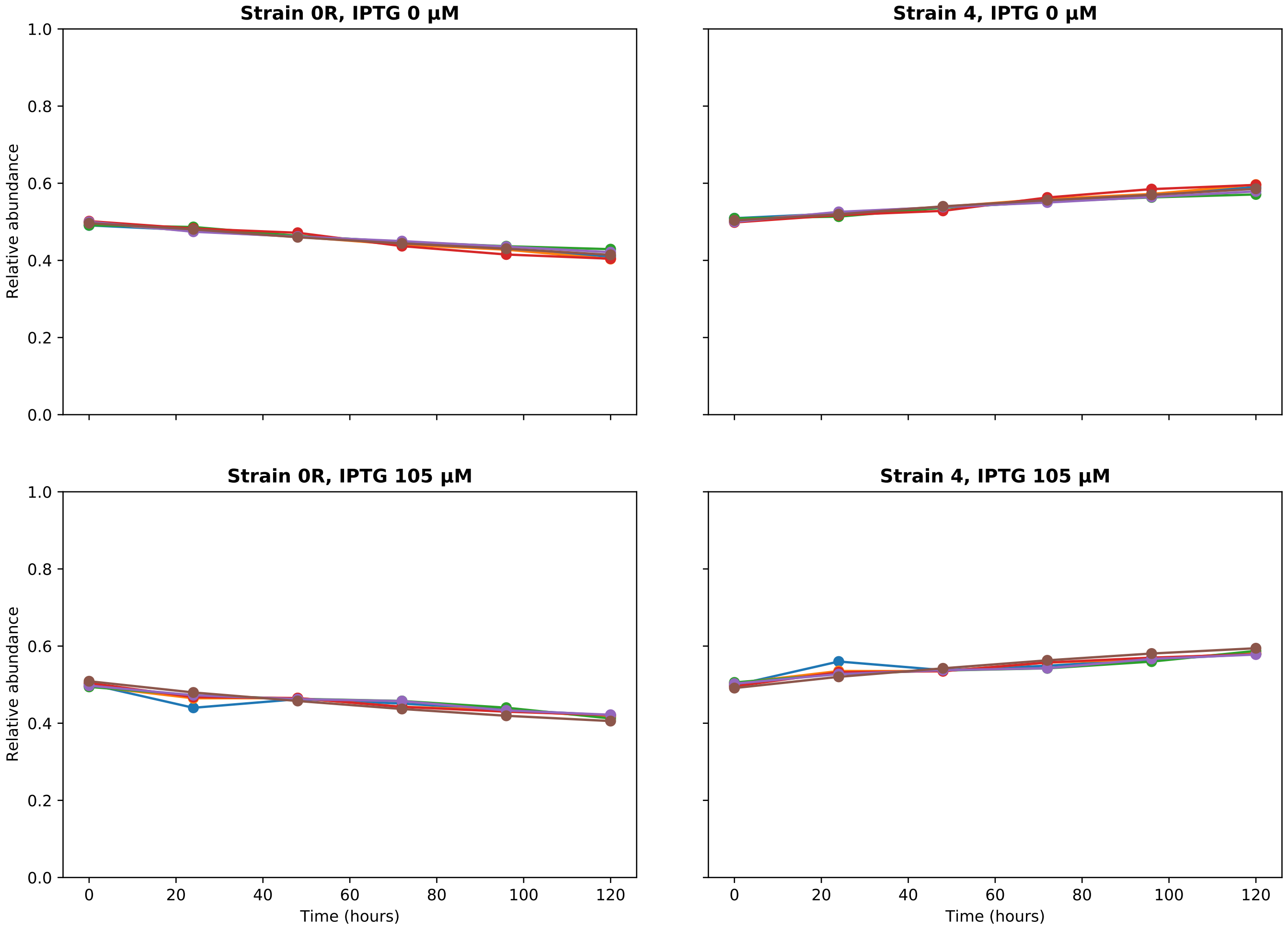

FIG. S.9: Competition assays between strains 0R and 4. These assays were done with the same experimental protocol shown in the Methods section (without adding ampicillin to the culture medium since strain 0R is not ampicillin resistant). As we can see, the plasmid containing ampicillin resistance carried by strain 4 confers a slight fitness advantage.

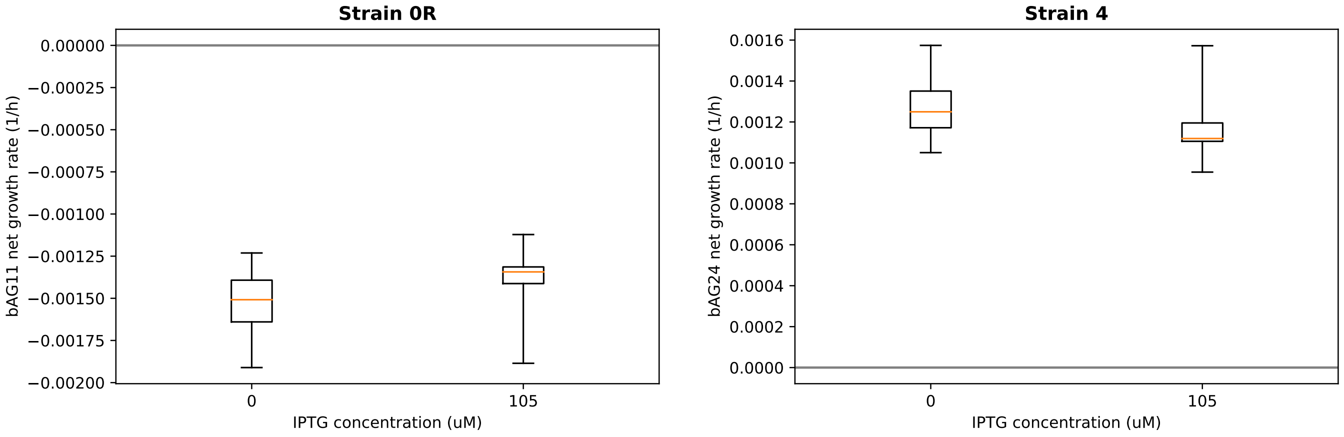

FIG. S.10: Boxplot of the growth rates of strains 0R and 4, computed by fitting the time series shown in Figure S.9

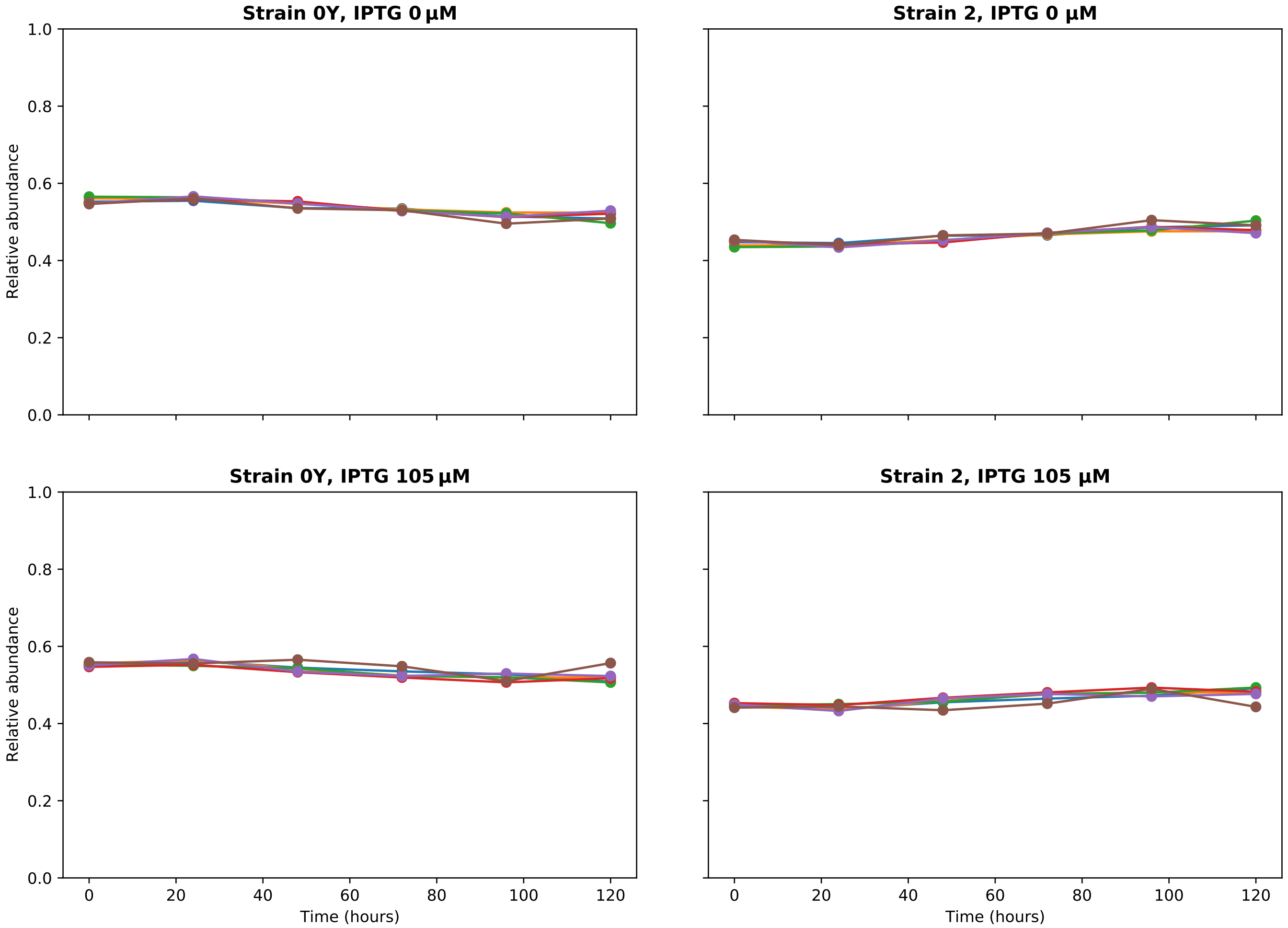

FIG. S.11: Competition assays between strains 0Y and 2. These assays were done with the same experimental protocol shown in the Methods section (without adding ampicillin to the culture medium since strain 0Y is not ampicillin resistant).

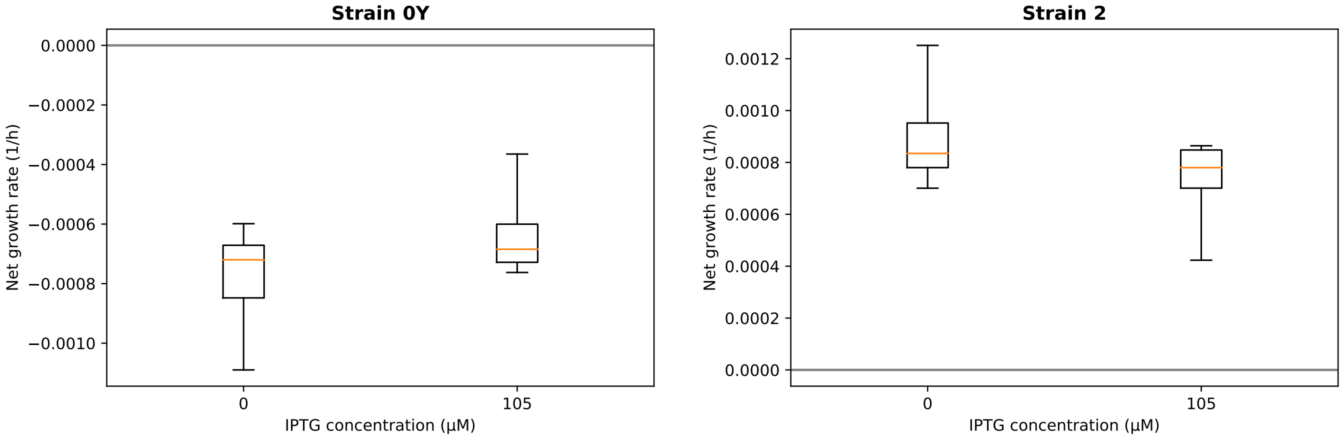

FIG. S.12: Boxplot of the growth rates of strains 0Y and 2, computed by fitting the time series shown in Figure S.11.

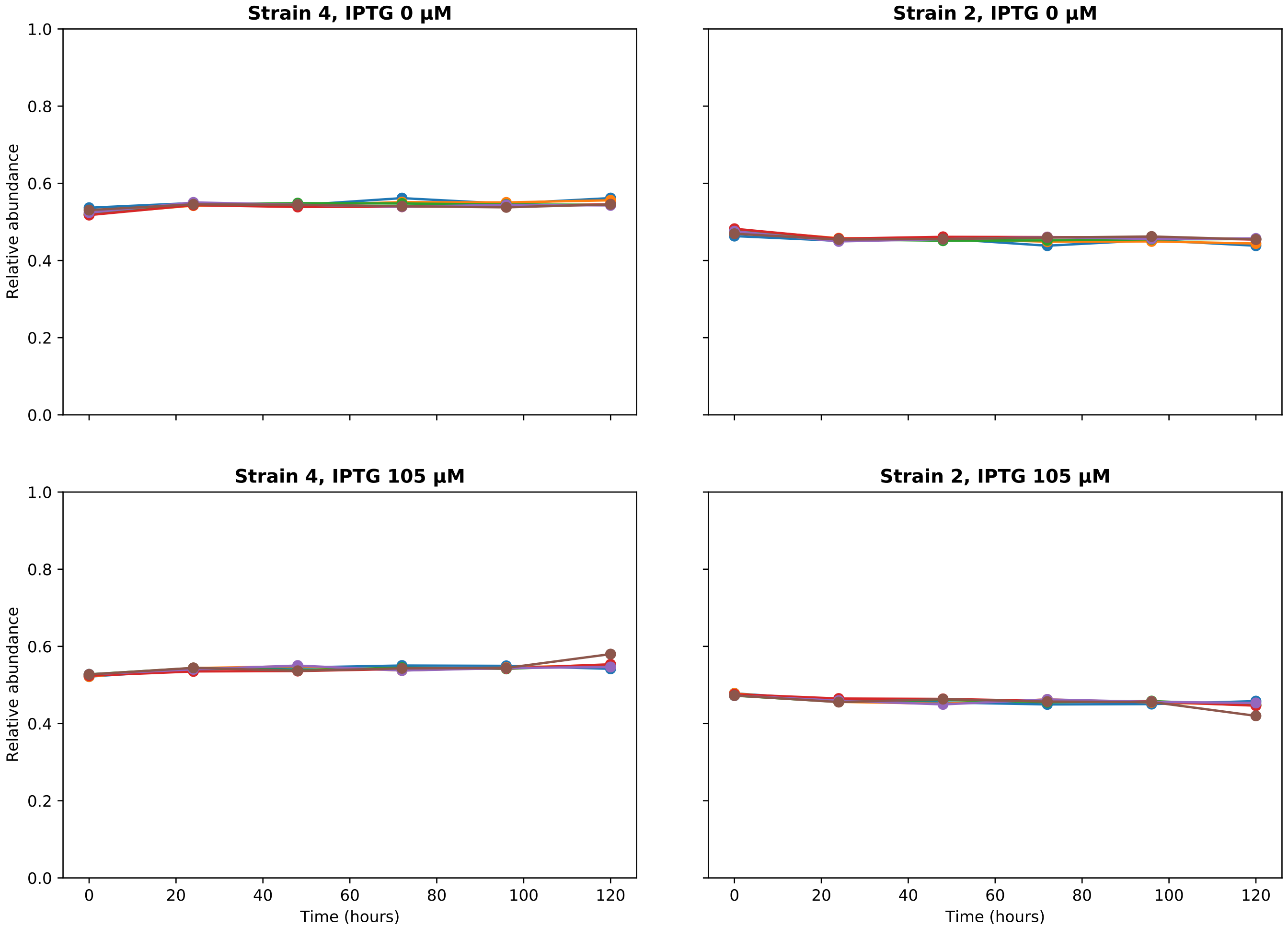

FIG. S.13: Competition assays between strains 4 and 2. These assays were done with the same experimental protocol shown in the Methods section.

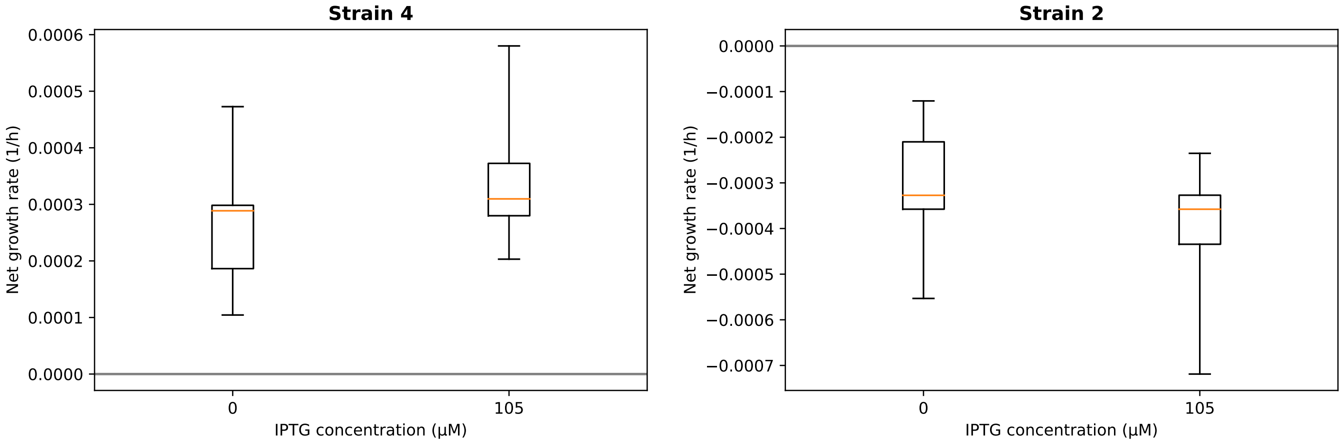

FIG. S.14: Boxplot of the growth rates of strains 4 and 2, computed by fitting the time series shown in Figure S.13

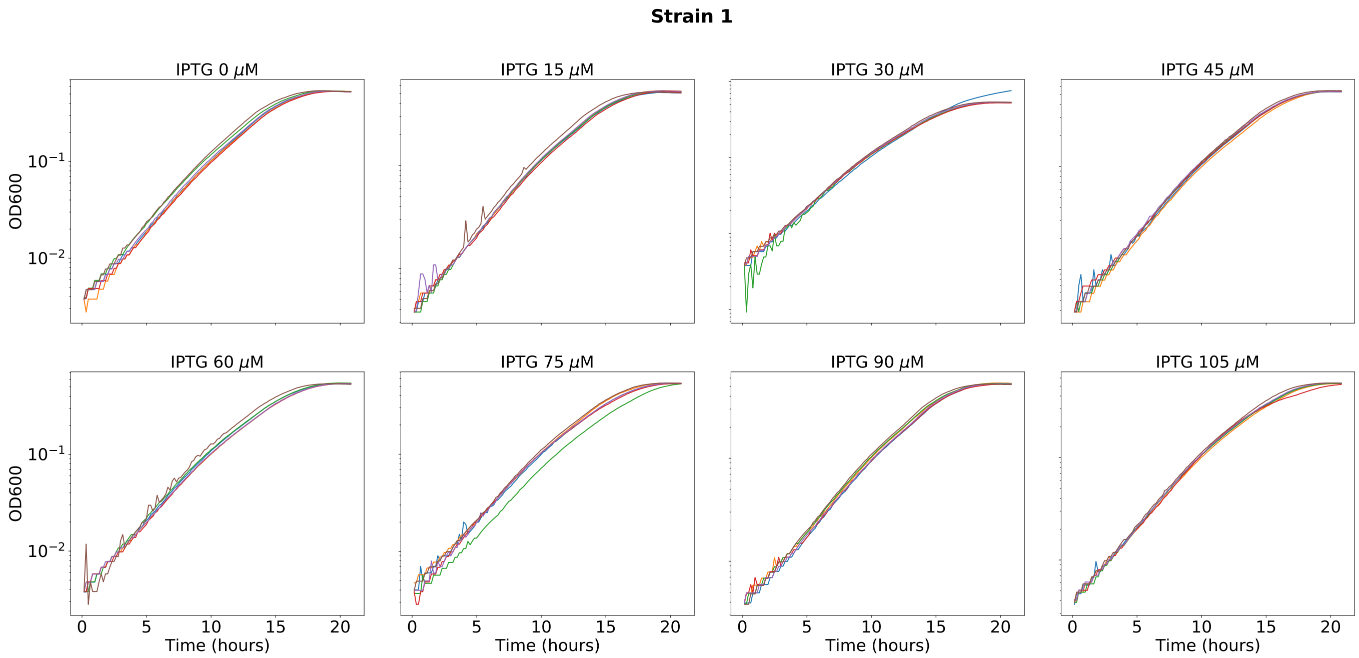

FIG. S.15: OD600 curves of strain 1 for different IPTG concentrations. These measures were obtained by preparing the strain with the same experimental protocol shown in the Methods section.

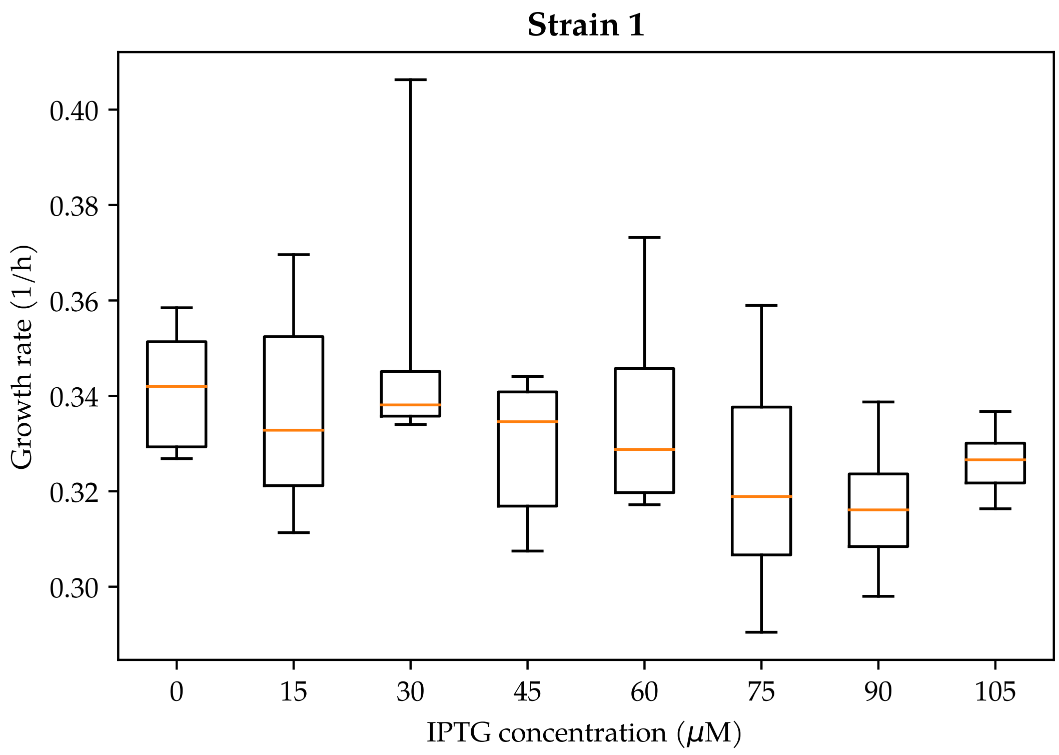

FIG. S.16: Boxplot of the growth rates of strain 1, obtained by fitting the curves in Figure S.15.

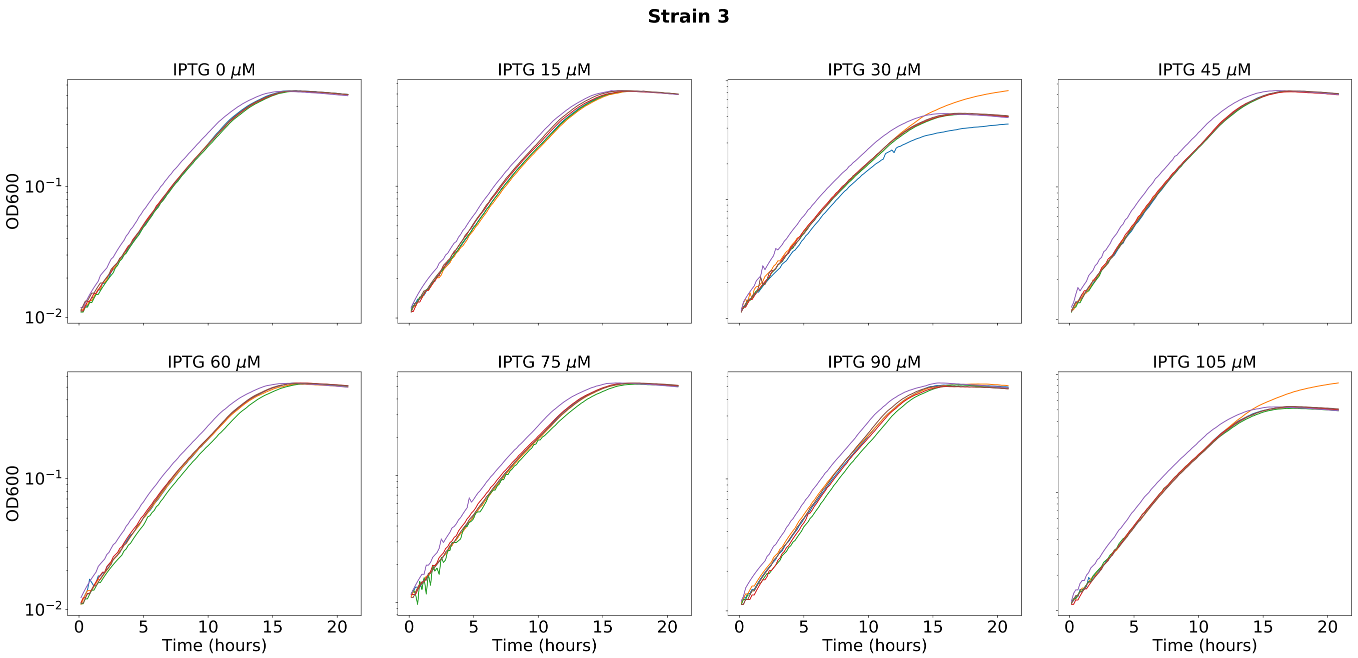

FIG. S.17: OD600 curves of strain 3 for different IPTG concentrations. These measures were obtained by preparing the strain with the same experimental protocol shown in the Methods section.

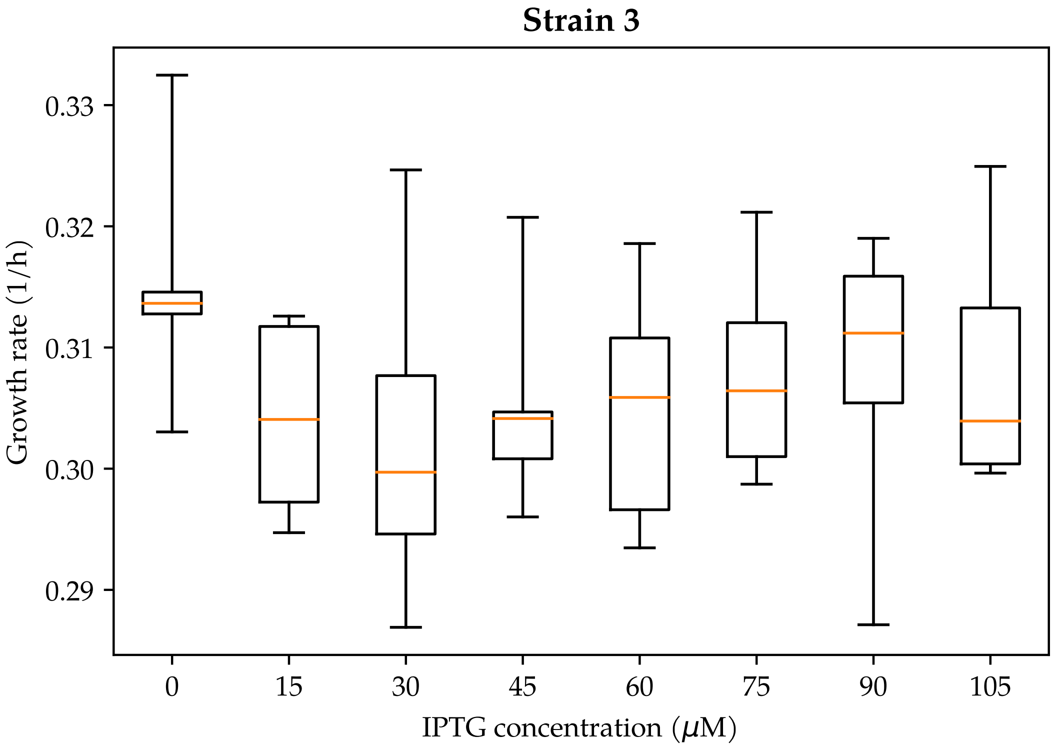

FIG. S.18: Boxplot of the growth rates of strain 3, obtained by fitting the curves in Figure S.17.

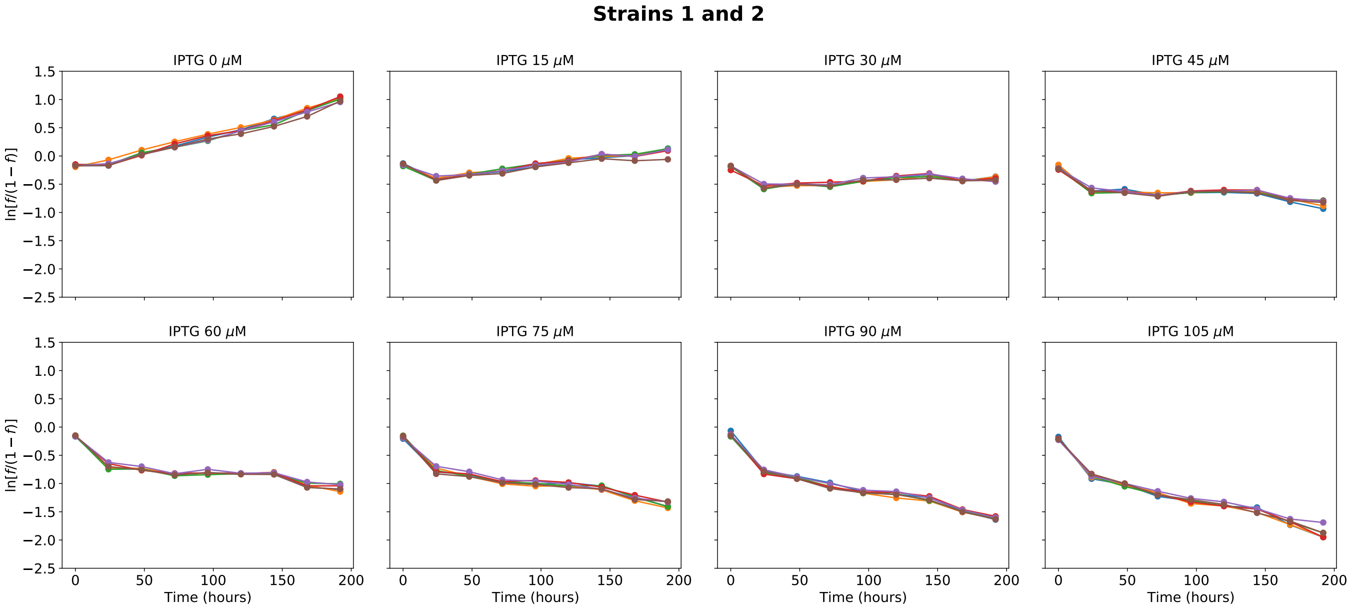

FIG. S.19: Time series of the values of $\ln f/(1-f)$ (with $f = m_1/(m_1+m_2)$ relative to the competition assay between strains 1 and 2. Each curve represent one of the six replicas of the treatment. The selective advantage $\mathcal{S}$ has been computed by fitting each of these curves with a linear function between day 1 and day 6 (i.e., between 24h and 144h).

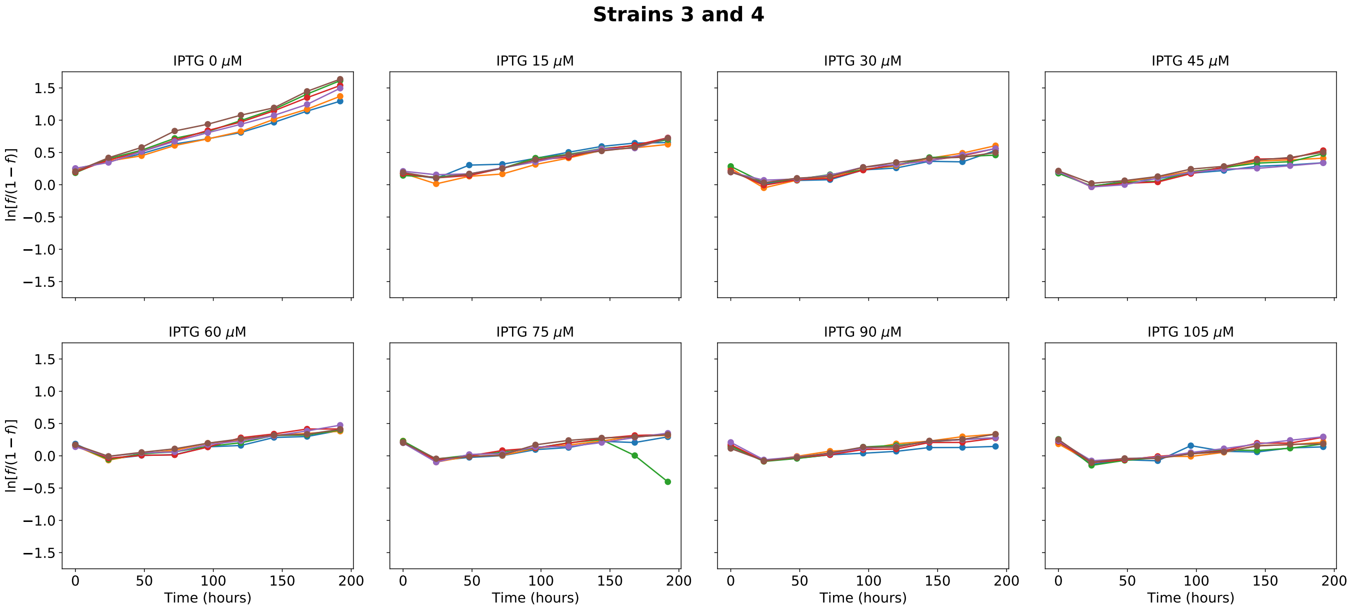

FIG. S.20: Time series of the values of $\ln f/(1-f)$ (with $f = m_3/(m_3+m_4)$ relative to the competition assay between strains 3 and 4. Each curve represent one of the six replicas of the treatment. The selective advantage $\mathcal{S}$ has been computed by fitting each of these curves with a linear function between day 1 and day 6 (i.e., between 24h and 144h).

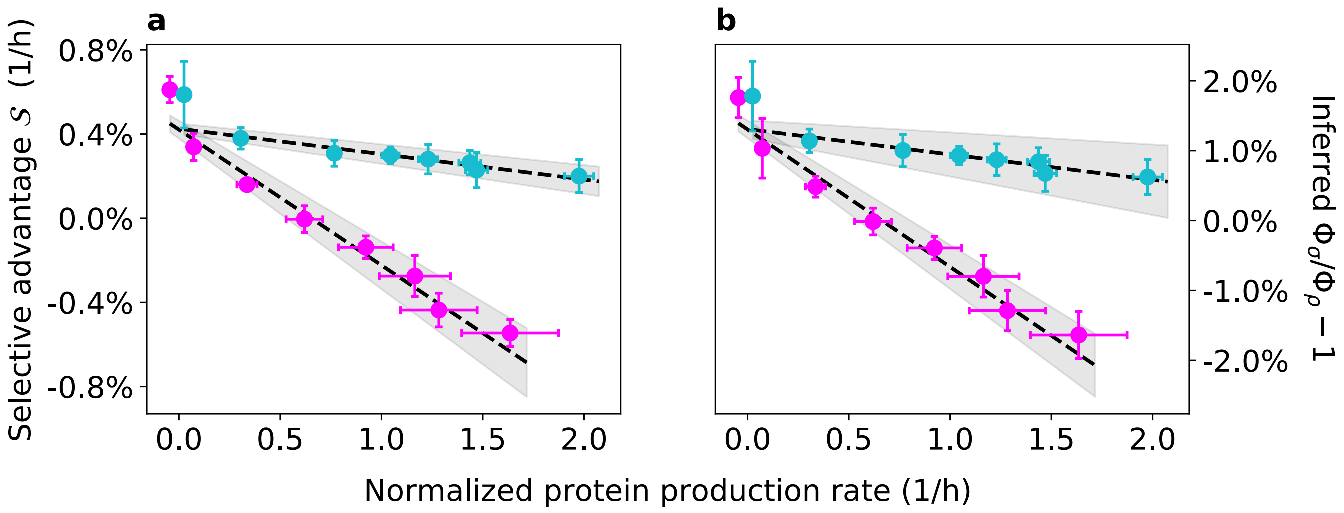

FIG. S.21: Results of the experiments (a) and inferred values of the ratios Φ_1/Φ_2 and Φ_3/Φ_4 (minus one), as shown in Figure 3 of the Main Text. In other words, magenta (cyan) points represent data of the experiment with strains 1 and 2 (3 and 4), error bars of the data points represent two standard deviations, and grey bands represent the 68% confidence interval of the linear fit). Differently from Figure 3, the linear fits shown here have been performed by including also the points at the lowest protein production rates (i.e., at 0 μM IPTG).
